# Comparison of Cyclic and Linear PEG Conjugates

**DOI:** 10.1021/acs.bioconjchem.4c00202

**Published:** 2024-05-29

**Authors:** Grace E. Kunkel, Qingyang Zhou, Joseph W. Treacy, Hayden R. Montgomery, Pedro Salas-Ambrosio, Austin D. Ready, Alexander M. Spokoyny, Kendall N. Houk, Heather D. Maynard

**Affiliations:** † Department of Chemistry and Biochemistry, University of California, Los Angeles, Los Angeles, California 90095, United States; ‡ California NanoSystems Institute, University of California, Los Angeles, Los Angeles, California 90095, United States

## Abstract

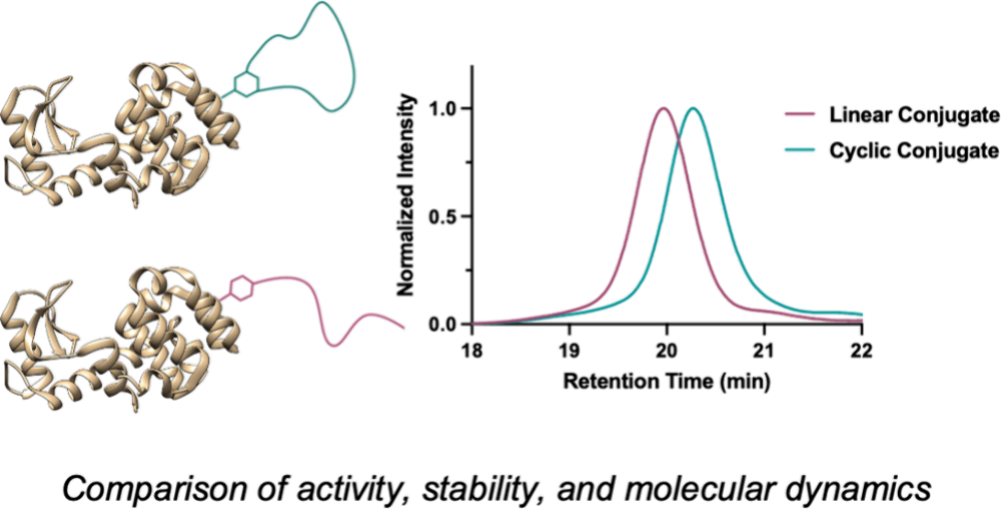

Bioconjugation of polymers to proteins is a method to impart improved stability and pharmacokinetic properties to biologic systems. However, the precise effects of polymer architecture on the resulting bioconjugates are not well understood. Particularly, cyclic polymers are known to possess unique features such as a decreased hydrodynamic radius when compared to their linear counterparts of the same molecular weight, but have not yet been studied. Here, we report the first bioconjugation of a cyclic polymer, poly(ethylene glycol) (PEG), to a model protein, T4 lysozyme, containing a single engineered cysteine residue (V131C). We compare the stability and activity of this conjugate with those of a linear PEG-T4 lysozyme analogue of similar molecular weight. Furthermore, we used molecular dynamics (MD) simulations to determine the behavior of the polymer–protein conjugates in solution. We introduce cyclic polymer–protein conjugates as potential candidates for the improvement of biologic therapeutics.

## Introduction

While researchers seek to improve therapeutic efficacy of drug treatments across several disease types, off-target effects often limit their advancement to the clinic and beyond.^1^ As a result, the number of protein-based drug products approved by the U.S. Food and Drug Administration (FDA) is steadily increasing, in part due to the specificity of their method-of-action.^2^ Protein biologics are a powerful class of therapeutics toward effective disease treatment; however, they are susceptible to degradation and clearance *in vivo*.^3,4^ Therefore, polymers are commonly used to provide stability and increased circulation times for biologics.^5,6^ Furthermore, polymers can also provide “stealth” properties for protein therapeutics that initiate undesired immunogenic responses.^7^ Currently, poly(ethylene glycol) (PEG) is the only polymer approved by the FDA for use in polymer-conjugated protein formulations. The FDA has approved over 30 PEGylated proteins that range in polymer size and linkage chemistry to tune properties such as circulation time and conjugation lability.^8^ Despite these successful examples, the conjugation of PEG to proteins has led to deleterious effects, such as contributing to vacuolization *in vivo* and inducing immunogenic responses as a result of the formation of anti-PEG antibodies.^9,10^ Although there has been extensive research using linear polymers other than PEG to conjugate to biologics,^11^ the effects of more complex polymer architectures on protein–polymer conjugate properties is largely limited to branched and brush polymers.^12^ Namely, branched and brush polymers are known to possess longer circulation times and high stability to proteolysis compared to their linear counterparts.^13,14^ Brush polymers also possess lower solution viscosity compared to linear analogues due to their elongated “rod-like” structures that align with solution flow direction.^15,16^ Low viscosity biologic formulations are likely to increase patient compliance as thinner needles can be used for injection. To date, linear and singly branched PEG polymers are the only polymer architectures available on the protein–polymer therapeutic market.^17^

Cyclic polymers are a macromolecular class known to have unique physical properties, such as a slower degradation profile and reduced hydrodynamic radius compared to their linear counterparts.^18^ These features make cyclic polymers an attractive modality for biomedical applications.^19^ Furthermore, most biologics are delivered via subcutaneous or intravenous injections, which are limited by injection volume (<1.5 mL).^20^ Therefore, biologics that necessitate a high dosage to reach efficacy must be highly concentrated formulations, which can lead to increased viscosity.^21^ In these cases, cyclic polymers that possess inherently reduced hydrodynamic radii compared to their linear counterparts may provide equal stabilizing effects while also imparting favorable physical properties to a biologic formulation, such as increased circulation times *in vivo*.

Herein, we describe the first example of a cyclic protein–polymer conjugate and compare it to a linear protein–polymer conjugate of the same size. For our model study, we synthesized linear and cyclic 2 kDa PEG Au(III) oxidative addition complexes and performed *S*-arylation of each substrate to the surface-exposed cysteine of T4 lysozyme V131C (T4L).^22−24^ We compared the conformation, stability, and activity of the resulting purified conjugates. Finally, we performed molecular dynamics simulations of these conjugates to examine and quantify the effect of polymer architecture on the protein–polymer conjugate behavior.

## Results and Discussion

### Synthesis of Cyclic and Linear 2 kDa PEG-Au(III) Reagents

One of the most significant barriers to the implementation of cyclic polymers in medicine is the challenge of their synthesis and purification.^18^ Linear contaminants are known to profoundly impact rheological properties,^25^ and batch-to-batch heterogeneity could preclude FDA approval. Moreover, polymers to be used in bioconjugation are typically modified at their termini. The lack of chain ends in cyclic polymers represents an additional complication to their synthesis for this application.

**Figure 1:**
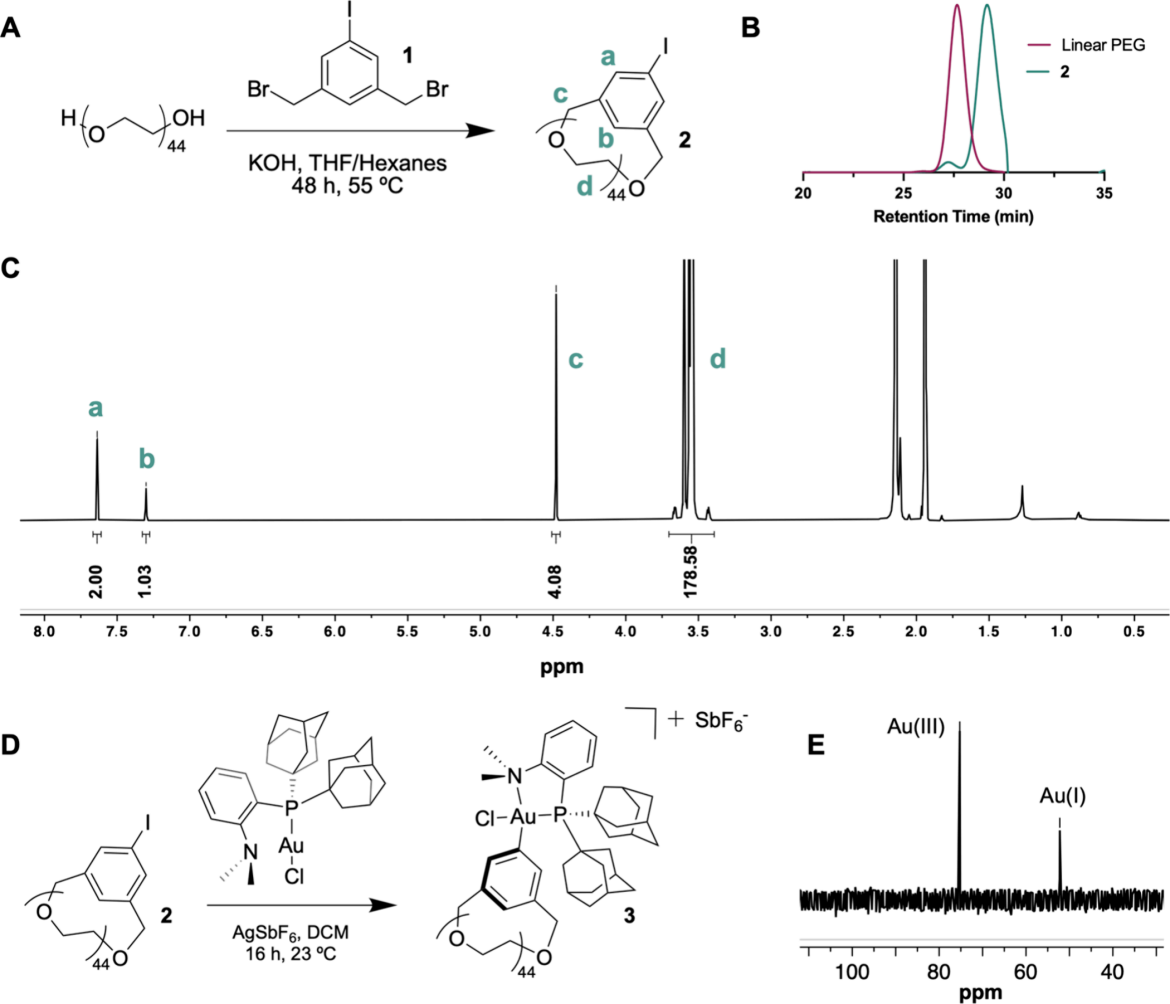
(A) Williamson ether synthesis of cyclic 2 kDa PEG-aryl iodide (**2**). (B) DMF SEC of linear 2 kDa PEG and **2**. (C) ^1^H NMR spectrum of **2** in CD_3_CN. (D) Oxidative addition of **2** with (Me-DalPhos)AuCl and AgSbF_6_ to yield **3**. (E) ^31^P{^1^H} NMR of **3** in CD_3_CN.

The Au(III)-mediated *S*-arylation strategy is well-suited to mitigate these synthetic challenges; the preparation of a cyclic PEG Au(III) oxidative addition complex requires an aryl iodide precursor, which is relatively ubiquitous in organic chemistry. Aryl iodides also provide a convenient orthogonal functional handle, which can be carried through multiple harsh chemical synthesis steps and selectively metalated with an Au(I) precursor at late stages.^26−29^ Therefore, we selected a bimolecular ring-closure strategy composed of a homodifunctional PEG diol and a difunctional benzyl bromide small molecule linker containing a sterically available aryl iodide (**1**). Accordingly, we used the commercially available dimethyl 5-iodoisophthalate and performed a reduction of the esters to the corresponding diol with NaBH_4_ and CaCl_2_, wherein the aryl iodide remained intact (SI Figures S1 and S2, Scheme S1). Subsequent bromination of both benzyl alcohol positions afforded **1**, which contains two benzyl bromides and a sterically available aryl iodide for further functionalization (SI Figures S3 and S4). Williamson ether synthesis between a commercial 2 kDa PEG and **1** afforded the cyclic polymer **2** (Figure 1A), which is >95% pure according to ^1^H NMR and analytical high performance liquid chromatography (Figure 1C, SI Figures S5 and S7).^30^ Specifically for the former, we observe aryl protons in the ^1^H NMR spectrum of **2** that possess integration ratios that correspond to a high level of cyclic polymer purity. As expected, the increased retention time of **3** compared to commercial 2 kDa PEG, as observed by DMF SEC, indicates that **2** possesses a smaller hydrodynamic radius (Figure 1B). In agreement with these observations, we also measured and calculated the intrinsic viscosity (η) of **2** and its linear mPEG (2 kDa)-aryl iodide analog to be 0.003 and 0.007 mL/mg, respectively (Figure S8). Next, we performed oxidative addition with (Me-DalPhos)AuCl, which afforded the bench-stable cyclic PEG-Au(III) oxidative addition complex **3** in good conversion and purity (Figure 1D, see SI for details). Notably, excess (Me-DalPhos)AuCl was present in the product (Figure 1E, SI Figure S10), but it has been shown previously that it does not have deleterious effects in the subsequent *S*-arylation step.^31^ Additionally, the linear 2 kDa mPEG Au(III) oxidative addition complex (**4**) was synthesized as previously described in order to compare the effects of the polymer architecture on the protein conjugation and its subsequent properties.^23^

### Preparation of PEG-T4L Conjugates

**Figure 2:**
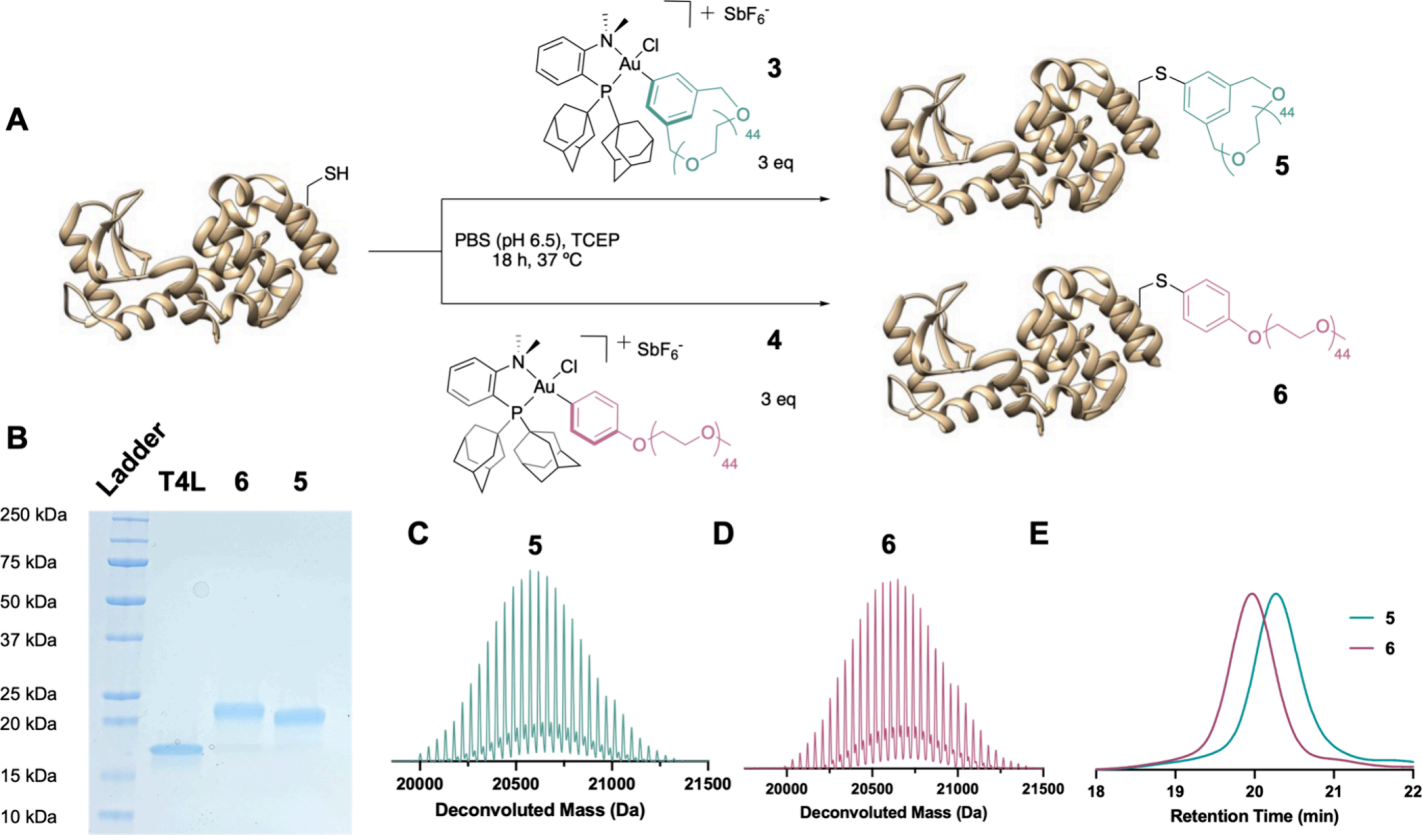
(A) Synthetic scheme representing T4L bioconjugation (PDB ID: 2HUK) to 2 kDa cyclic PEG (**3**) and 2 kDa linear mPEG (**4**), resulting in conjugates **5** and **6**, respectively. (B) Coomassie-stained SDS-PAGE gel of T4L, **6**, and **5**. By ImageJ optical densitometry, **6** and **5** are 96% and 98% converted from the T4L starting material, respectively. (C) LCMS of **5**. Calculated mass is 20661.6 Da, and observed mass is 20662.3 Da. (D) LCMS of **6**. Calculated mass is 20693.6 Da, and observed mass is 20694.7 Da. (E) SEC FPLC spectrum for **5** and **6**.

To prepare singly PEGylated T4L, a mutant T4 lysozyme containing one surface-exposed cysteine (V131C) was expressed (SI Figure S10).^32,33^ Then, T4L was treated with 4 equiv of tris(2-carboxyethyl)phosphinehydrochloride (TCEP·HCl) for 1 h at 37 °C to reduce the dimeric form of the protein formed by intermolecular disulfide bonds. The equivalents and temperature of the TCEP·HCl reduction were optimized to produce quantitative T4L monomer (SI Figure S12). As shown in previous work, TCEP·HCl did not negatively affect the *S*-arylation reaction and therefore did not necessitate removal.^23^ Next, three equivalents of **3** and **4** were each incubated at 37 °C with 70 μM T4L in PBS (pH 6.5) for 18 h to produce cyclic PEG-T4L (**5**) and linear mPEG-T4L (**6**), respectively (Figure 2A). PEG equivalents, reaction time, and reaction temperature were screened (SI Figures S13 and S14) to produce nearly quantitative conversion to conjugates **5** and **6** (98% and 96%, respectively) as observed by SDS-PAGE and determined by ImageJ optical densitometry (Figure 2B). Experiments at ambient temperature (23 °C) with all other variables held constant also resulted in high conversion to **5** and **6** (SI Figure S15, 84% and 80%, respectively), suggesting that this method can also be used at lower temperatures. It is important to note that kinetics of this *S*-arylation reaction are far slower than that of previous Au(III)-mediated PEGylation in a DARPin protein system,^23^ which we hypothesize is due to the different local environment of the Cys residue in the protein. However, this still needs to be studied. Following the *S*-arylation, **5** and **6** were purified by size exclusion fast protein liquid chromatography (SEC FPLC) to remove excess PEG reagent and Au(I) byproducts (see SI for details). Liquid chromatography–mass spectrometry (LCMS) of **5** and **6** produced deconvoluted mass values that correspond to each respective expected value (Figure 2C and D). As cyclic ethers are known to effectively coordinate metal cations,^34^ we aimed to ensure that this method was sufficient to remove excess Au. Accordingly, inductively coupled plasma optical emission spectroscopy (ICP-OES) was performed, and it was determined that <50 ppb Au remained following the SEC FPLC process for both **5** and **6** (see SI for details).

As previously seen in their polymeric counterparts (Figure 1B), the protein–polymer conjugate **5** possesses a longer SEC FPLC retention time than that of **6** (Figure 2E). Interestingly, this trend can also be observed by a smaller gel-shift of **5** compared to that of **6** in SDS-PAGE (Figure 2B). Therefore, as expected,^35^ the smaller hydrodynamic radius of cyclic PEG compared to its linear counterpart is shown to translate to an overall smaller hydrodynamic radius of the cyclic polymer–protein conjugate. As the hydrodynamic radius is known to directly correlate to viscosity, cyclic polymer–protein conjugates will likely result in a less viscous biologic formulation.^36^

### Characterization of PEG-T4L Conjugates

**Figure 3:**
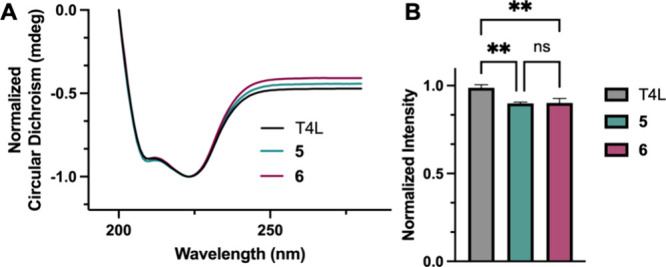
(A) Normalized CD spectrum of T4L, **5**, and **6** at 23 °C showing no observable difference in helicity. (B) Lysozyme activity fluorescence assay of T4L, **5**, and **6**. *n* = 3 for each group. An ordinary one-way ANOVA statistical analysis was performed. **p < 0.005. ns = not significantly different.

To determine whether the polymer architecture within a bioconjugate affects the secondary structure of the protein, T4L, **5**, and **6** were analyzed by circular dichroism (CD). In PBS (pH 6.5) at 23 °C, we see no observable difference in helicity for T4L or its PEGylated conjugates (Figure 3A). Characteristic local minima are observed in each trace at 209–210 and 223 nm, which is consistent with the protein and its conjugates adopting an alpha helical structure. Therefore, we conclude that there is no conformational difference for the protein imparted by varying the PEG architecture conjugated to T4L.

To compare the stabilizing effects of cyclic and linear PEG for T4L, CD was used to determine the experimental melting temperature (*T*_m_) of T4L, **5**, and **6** (SI Figure S19).^37^ Using a temperature ramp from 20 to 100 °C and monitoring the relative helicity at 222 nm, we observe a *T*_m_ for T4L at 56.8 °C. **5** and **6** were determined to have a *T*_m_ of 63.2 and 62.6 °C, respectively. Consequently, we conclude that cyclic PEG conjugates have the potential to stabilize T4L to a similar extent as the linear counterpart.

Modification of enzymes with PEG can have deleterious effects on their activity, often due to changes in electrostatic effects on the protein surface, modification of the protein conformation, and/or interaction of the polymer with the active site.^38,39^ As we previously observed *vide supra*, there is no significant change in the secondary structure of T4L after PEGylation with either **5** or **6**, though the influence on the activity of T4L was still unknown. To investigate, we compared the activity of T4L, **5**, and **6** through the cell lysis of FITC-labeled Gram-positive *Micrococcus luteus* as monitored by an EnzChek lysozyme activity assay (see SI for details). Comparing the PEG conjugates (**5** and **6**) with unmodified T4L, we observe a statistically significant difference between the unmodified protein and each conjugate, wherein the conjugates are approximately 10% less active (Figure 3B). This is consistent with previous literature reports where mutations distal to the active site such as in this case have a lower effect on disruption of T4L activity.^40^ However, there is no statistical significance between **5** and **6**, suggesting that the conformation of the cyclic polymer chain does not negatively affect the protein’s activity compared to its linear counterpart at this molecular weight (2 kDa).

**Figure 4:**
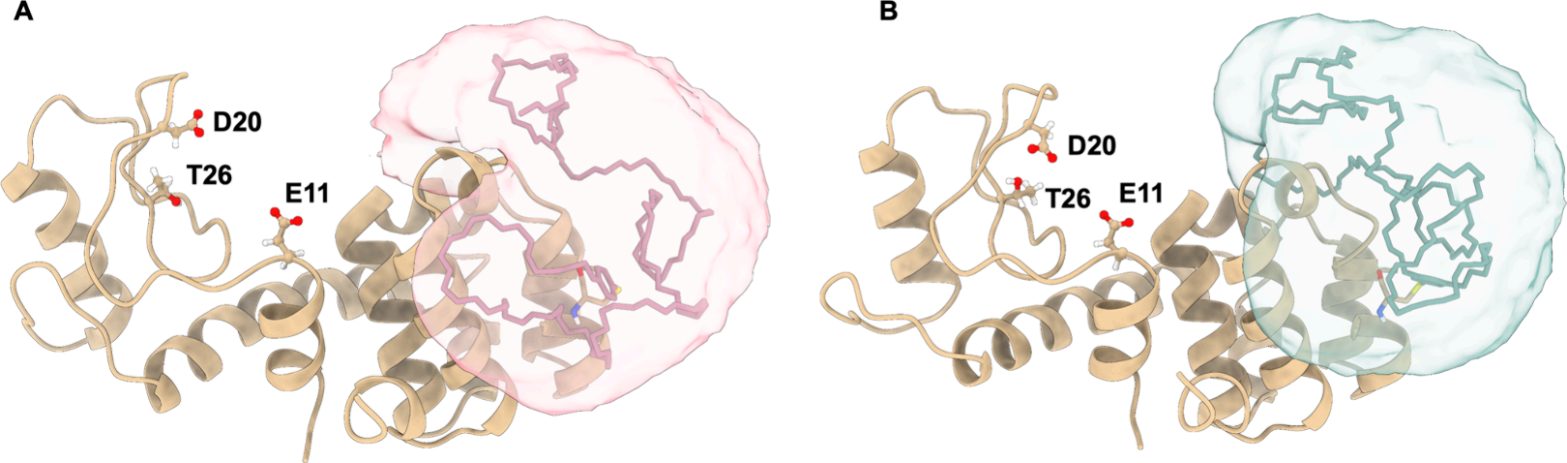
Average polymer distribution isosurfaces resulting from three independent 1000 ns molecular dynamic simulations for (A) **6** and (B) **5**. Although there is no protein conformational difference induced by the polymer chain architecture, the cyclic PEG interacts less frequently with the T4L binding site compared to its linear counterpart.

To understand the behavior of the conjugate in solution, we performed three independent 1000 ns molecular dynamics simulations for conjugates **5** and **6** (see SI for details and simulation videos, Figure 4). Similar to our CD experiments, there was no significant conformational difference for T4L induced by the cyclic and linear polymer chains. However, we observe that the linear polymer of **6** interacts with the active site (E11-D20-T26) of T4L in approximately 5% of the simulations, whereas the cyclic polymer of **5** interacts with the active site in <0.1% of the simulations (See SI Figures S22–S28). Although these results suggest similar behavior of the polymer chains in solution, a conformationally restricted cyclic polymer may prove beneficial in the preparation of protein–polymer conjugates wherein the conjugation site is nearer the active site of the enzyme. Additionally, we also hypothesize that utilization of a cyclic scaffold could be advantageous for longer polymers, where interaction with distant active sites is spatially more likely.

## Conclusion

Bioconjugation of polymers to proteins is a decades-long practice to impart the desired functionality onto biologics. To the best of our knowledge, this report represents the first example of the preparation and biophysical characterization of a cyclic polymer–protein conjugate. Herein, we describe a straightforward method (three synthetic steps) to prepare a cyclic PEG containing a bioconjugation handle with minimal linear polymer contaminants. We observed that a cyclic polymer–protein conjugate possessed a smaller hydrodynamic radius compared to its linear counterpart, despite having equal protein conformation, stability effects, and enzyme activity. We believe that the implementation of cyclic polymers could have a substantial impact on the rheological properties of protein–polymer bioconjugate formulations, which will be studied in the future. We recognize the significant challenge posed by introducing new polymer architectures to clinical settings, from both financial and regulatory perspectives. Nevertheless, this work highlights the cyclic polymer scaffold as an emerging modality of bioconjugation and stresses the need for continued exploration of the polymer architecture for the improvement of biologic therapeutics.

## Data Availability

Primary data for this work can be found at the following Web site address: https://github.com/gkunkel19/Kunkel-et.al.-2024

## Supporting Information Available

Experimental details, NMR spectra, characterization, computational details (PDF)
Molecular dynamics simulations (MP4)
Molecular dynamics simulations (MP4)
