# Supplementary material for: Comparison of Cyclic and Linear PEG Conjugates
Source: Bioconjug Chem. 2024 May 29;35(6):744–9. doi: 10.1021/acs.bioconjchem.4c00202 (PMC11191396; doi:10.1021/acs.bioconjchem.4c00202)
Supplement: Supplementary file 1 — bc4c00202_si_001.pdf [file bc4c00202_si_001.pdf]

Supporting Information

# Comparison of Cyclic and Linear PEG Conjugates

Grace E. Kunkel,[1] Qingyang Zhou,[1] Joseph W. Treacy,[1] Hayden R. Montgomery,[1] Pedro Salas-Ambrosio,[1,2] Austin D. Ready,[1] Alexander M. Spokoyny,[1,2] Kendall N. Houk,[1,2] Heather D. Maynard[1,2]*

Affiliations:
[1]Department of Chemistry and Biochemistry, University of California, Los Angeles, Los Angeles California, 90095, United States.

[2]California NanoSystems Institute, University of California, Los Angeles, Los Angeles, California 90095, United States.

*Correspondence to maynard@chem.ucla.edu.

# Table of Contents

**Table of Contents**

*General Experimental Information* ............ *3*

*Synthesis of (Me-DalPhos)AuCl Reagent* ............ *5*

*Synthesis of Cyclic and Linear PEG Reagents* ............ *6*

**Synthesis of (5-iodo-1,3-phenylene)dimethanol (7)** ............ **6**

**Synthesis of 1,3-bis(bromomethyl)-5-iodobenzene (1)** ............ **9**

**Synthesis of Cyclic PEG (2 kDa)-Aryl Iodide (2)** ............ **11**

**Synthesis of Cyclic PEG (2 kDa) [(Me-DalPhos)AuCl][$SbF_6$] (3)** ............ **14**

**Synthesis of Linear PEG (2 kDa) [(Me-DalPhos)AuCl][$SbF_6$] (4)** ............ **17**

*T4 Lysozyme V131C (T4L) Protein Expression* ............ *18*

*Preparation of T4L-PEG Conjugates* ............ *20*

**General Experimental Information** ............ **20**

**T4L-PEG Conjugation Procedure: General Conditions** ............ **21**

**T4L Reduction TCEP·HCl Equivalent Screen** ............ **21**

**PEG Equivalent and Temperature Screen** ............ **22**

**Extended Time Screen** ............ **22**

*Characterization of PEGylated-T4 Lysozyme Conjugates* ............ *26*

**Circular Dichroism (CD) and Stability Assessment** ............ **26**

**Activity Assay** ............ **27**

**ICP-OES Analysis** ............ **29**

**Molecular Dynamics Simulations** ............ **30**

*References* ............ *34*

# General Experimental Information

Unless otherwise stated, all materials were purchased and used as received from Fisher Scientific, Combi-Blocks, Alfa Aesar, Oakwood Chemicals, or Sigma Aldrich. Silver hexafluoroantimonate ($AgSbF_6$) was stored in a glovebox maintained under a nitrogen atmosphere prior to use.

NMR spectra were recorded on the following: AV400 Bruker spectrometer at 400 ($^1$H) and 121 MHz ($^{31}$P{$^1$H}); AV300 Bruker spectrometer at 300 ($^1$H) and 75 MHz ($^{13}$C); NEO600 Bruker spectrometer at 600 ($^1$H) and 243 ($^{31}$P{$^1$H}) MHz. Spectra are reported in δ (parts per million) relative to residual proteo-solvent signals for $^1$H and $H_3PO_4$ (δ 0.00 ppm) for $^{31}$P{$^1$H}. The following abbreviations were used to explain multiplicities: s = singlet, d = doublet, t = triplet, q = quartet, m = multiplet. Deuterated solvents were purchased from Cambridge Isotope Laboratories and used as received for all NMR experiments. Anhydrous DCM was prepared *via* distillation over calcium hydride and stored under a nitrogen atmosphere. Anhydrous THF was prepared using an activated alumina column and stored under an argon atmosphere.

Column chromatography was performed on a Biotage Isolera One 3.0 autocolumn instrument using KP-Sil high-performance columns repacked using Silicycle silica (P60, particle size 40–63 µm, column sizes described in experimental). TLC was performed using Millipore Sigma silica plates (60F-254) using short-wave UV light as visualizing agents. Electrospray ionization (ESI) mass spectra were obtained using an Agilent 6530 QTOF-ESI in tandem with a 1260 Infinity LC. DART mass spectra were obtained using a Thermo Exactive Plus Orbitrap with IonSense ID-CUBE DART source.

Analytical reverse-phase high performance liquid chromatography (HPLC) was carried out on a Agilent 1260 Infinity II HPLC system equipped with an autosampler and a UV detector using a Poroshell 120 2.7 µm C18 120 Å column (Analytical: 2.7 µm, 4.6 × 100 mm) with monitoring at λ = 220 and 280 nm and with a flow rate of 0.8 mL/min. Preparative HPLC purification was carried out on an Agilent 1290 Infinity II liquid chromatography system equipped with a UV detector using a Luna 5 µm C18 100 Å column (Preparatory: 5 µm, 250 × 21.2 mm) with monitoring at λ = 214 and 254 nm and with a flow rate of 20 mL/min. Individual gradients for each product purified *via* reverse phase HPLC are specified in their respective procedures.

DMF Size Exclusion Chromatography (SEC)/Gel Permeation Chromatography (GPC) was conducted on an Agilent 1260 Infinity II high performance liquid chromatography (HPLC) system with a Wyatt Optilab (RI and MALS detection), one Polymer Laboratories PLgel guard column, and two Polymer Laboratories PLgel 5 µm mixed D columns. The eluent was DMF (HPLC Grade, 99.7+%, Thermo Scientific Chemicals) containing LiBr (0.1 M) at 40 °C (Flow rate: 0.6 mL/min). Molecular weight information was determined for data collected using a PMMA (Agilent Technologies, EasiVial PMMA, preweighed calibration kit) conventional calibration analysis.

Intrinsic viscosity measurement. Polymer solutions were prepared from a stock solution of 100 mg/mL in water at different concentrations. Then, the viscosity was measured using the RheoSense HVROC-L Portable Viscometer Control Unit (USA) at 25 °C and using a constant shear rate of 8300 $s^{-1}$. The intrinsic viscosity of linear and cyclic PEG was determined using the Huggins

equation by plotting the specific viscosity over concentration versus the concentration according to the following equation:

$$\frac{\eta_{sp}}{c} \cong [\eta] + k_H[\eta]^2 c$$

Where $\eta_{sp}$ is the specific viscosity, $c$ is the concentration, $[\eta]$ is the intrinsic viscosity, $k_H[\eta]^2$ is the second-order coefficient which contains the Huggins constant ($k_H$).

# Synthesis of (Me-DalPhos)AuCl Reagent

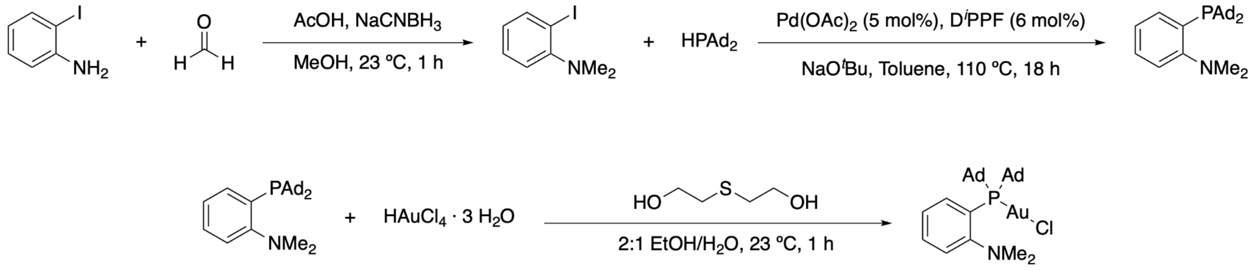


**Scheme S1.** Synthesis of (Me-DalPhos)AuCl.

The (Me-DalPhos)AuCl reagent was synthesized as reported previously.[1,2] Briefly, 2-iodoaniline was methylated using formaldehyde under reductive amination conditions. Next, the resulting 2-iodo-N,N-dimethylaniline product underwent Pd-catalyzed cross-coupling to produce Me-DalPhos (2-(diadamantylphosphinyl)-N,N-dimethylaniline).[3] Finally, this P,N ligand was metallated with chloroauric acid trihydrate and thiodiethanol to produce the (Me-DalPhos)AuCl reagent. All spectra match those of literature values.

# Synthesis of Cyclic and Linear PEG Reagents

## Synthesis of (5-iodo-1,3-phenylene)dimethanol (7)

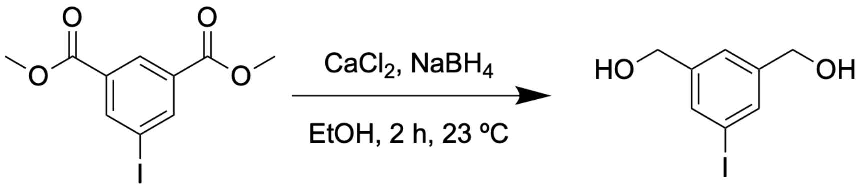


Dimethyl 5-iodoisophthalate (1.0 g, 1 eq, 3.1 mmol) was dissolved in ethanol (10 mL) in a round bottom flask charged with a stir bar. The reaction was brought to 0 ºC and sodium borohydride (485 mg, 4.1 eq, 12.8 mmol) was added slowly. Next, calcium chloride (728 mg, 2.1 eq, 6.6 mmol) in an additional 10 mL ethanol was added slowly. The reaction was allowed to warm to 23 ºC and stirred for 2 hours. Ethanol was removed under reduced pressure and the reaction was diluted with water. The crude product was extracted with ethyl acetate twice, dried over anhydrous $MgSO_4$, filtered, and concentrated under reduced pressure. The product was purified using column chromatography with a 0-10% gradient of methanol against DCM. The product was isolated as a white solid (740 mg, 2.8 mmol, 90% yield).

**Physical State:** White solid
**TLC (UV):** 0.67 (9:1 DCM-MeOH)
**$^1$H NMR (500 MHz, $CD_3CN$):** δ 7.59 (s, 2H), 7.29 (s, 1H), 4.66 – 4.23 (m, 4H), 3.27 (t, *J* = 6.0 Hz, 2H).
**$^{13}$C NMR (126 MHz, $CD_3CN$):** δ 145.75, 135.00, 125.21, 94.53, 63.77.
**HRMS (DART):** $[M-H]^-$ calculated for $C_8H_8IO_2^-$ 262.9574, observed 262.9576

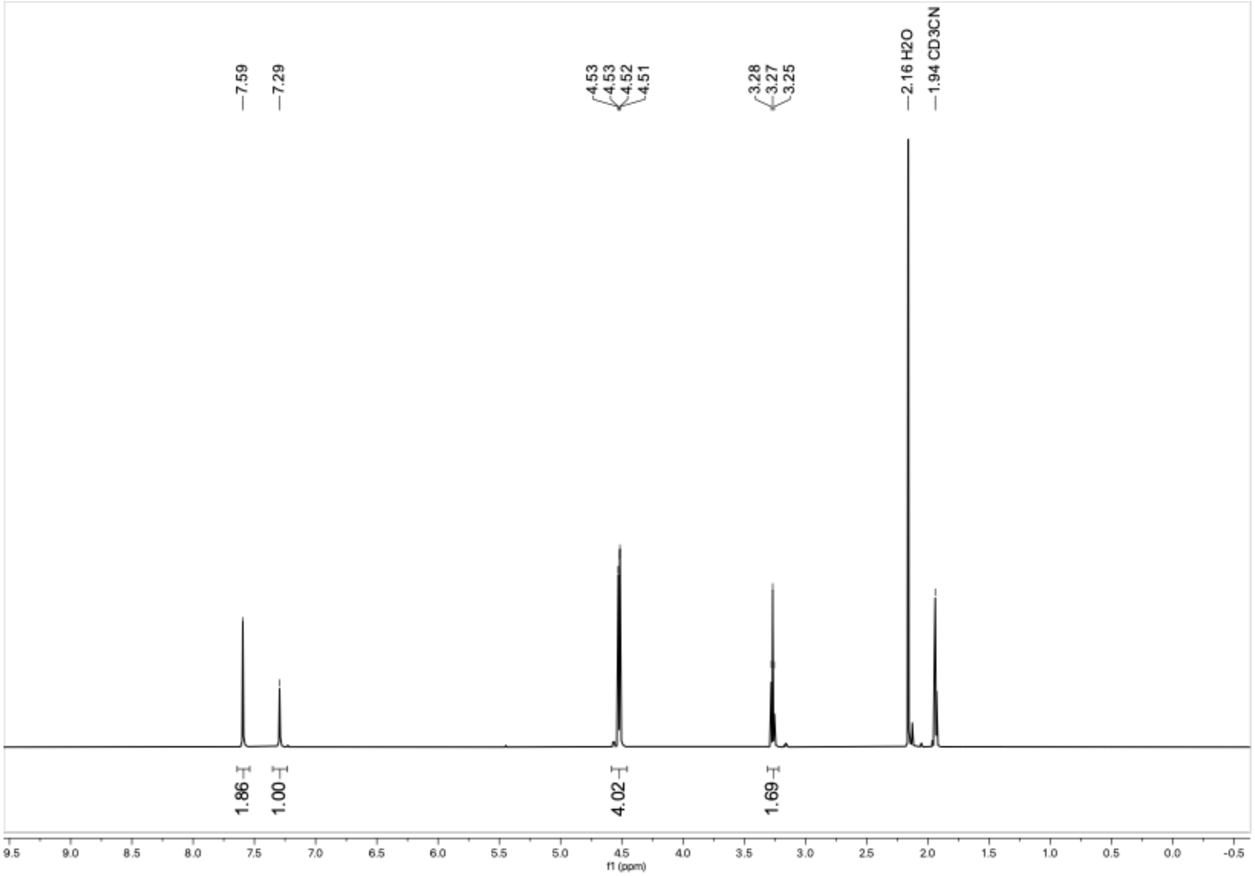


**Figure S1.** $^{1}$H NMR of (5-iodo-1,3-phenylene)dimethanol (**7**) in $CD_3CN$ at 23 ºC.

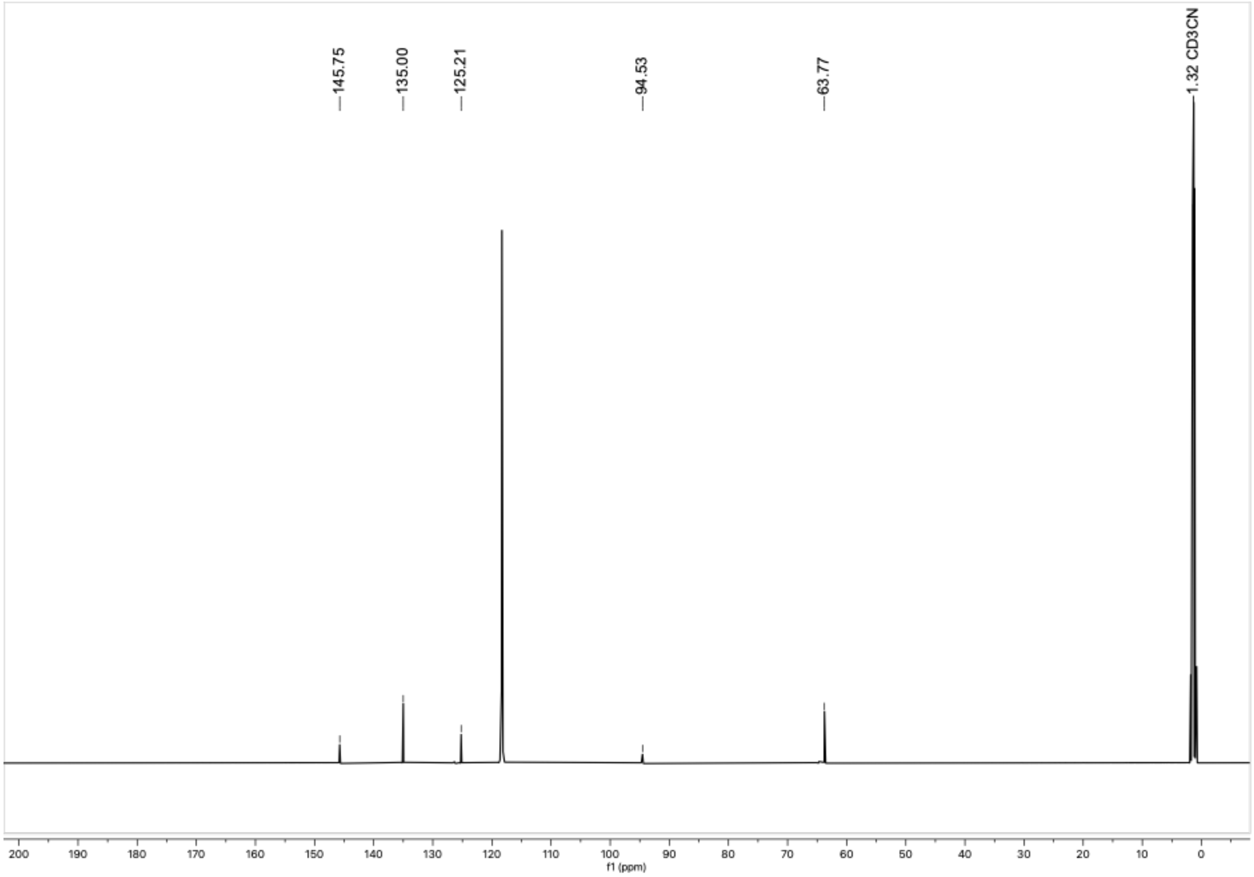

**Figure S2.** $^{13}$C NMR of (5-iodo-1,3-phenylene)dimethanol (**7**) in $CD_3CN$ at 23 ºC.

## Synthesis of 1,3-bis(bromomethyl)-5-iodobenzene (1)

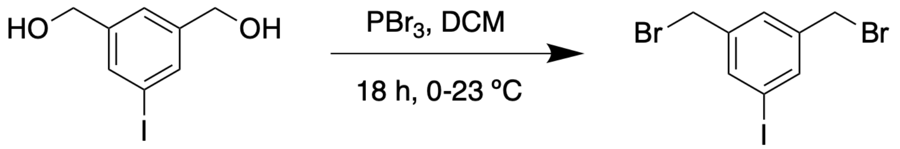


A flame dried two-neck round bottom flask was placed under argon atmosphere. (5-iodo-1,3-phenylene)dimethanol (500.0 mg, 1 eq, 1.9 mmol) was added with 10 mL anhydrous DCM. Next, the reaction was cooled to 0 ºC and phosphorus tribromide in DCM (2.1 g, 7.6 mL, 1.0 molar, 4 eq, 7.6 mmol) was added slowly. Finally, the reaction was warmed to 23 ºC and stirred for 18 hours. The crude product was concentrated under pressure and purified using column chromatography (0-100% ethyl acetate against hexanes). The product was isolated as a white solid (460 mg, 1.2 mmol, 62% yield).

**Physical State:** White solid
**TLC (UV):** 0.53 (9:1 Hexanes-EtOAc)
**$^1$H NMR (500 MHz, $CD_3CN$):** δ 7.75 (s, 1H), 7.49 (s, 1H), 4.49 (s, 4H).
**$^{13}$C NMR (126 MHz, $CD_3CN$):** δ 142.19, 138.72, 130.29, 94.50, 32.57.
**HRMS (DART):** $[M]^+$ calculated for $C_8H_7Br_2I^+$ 387.7959, observed 387.7945.

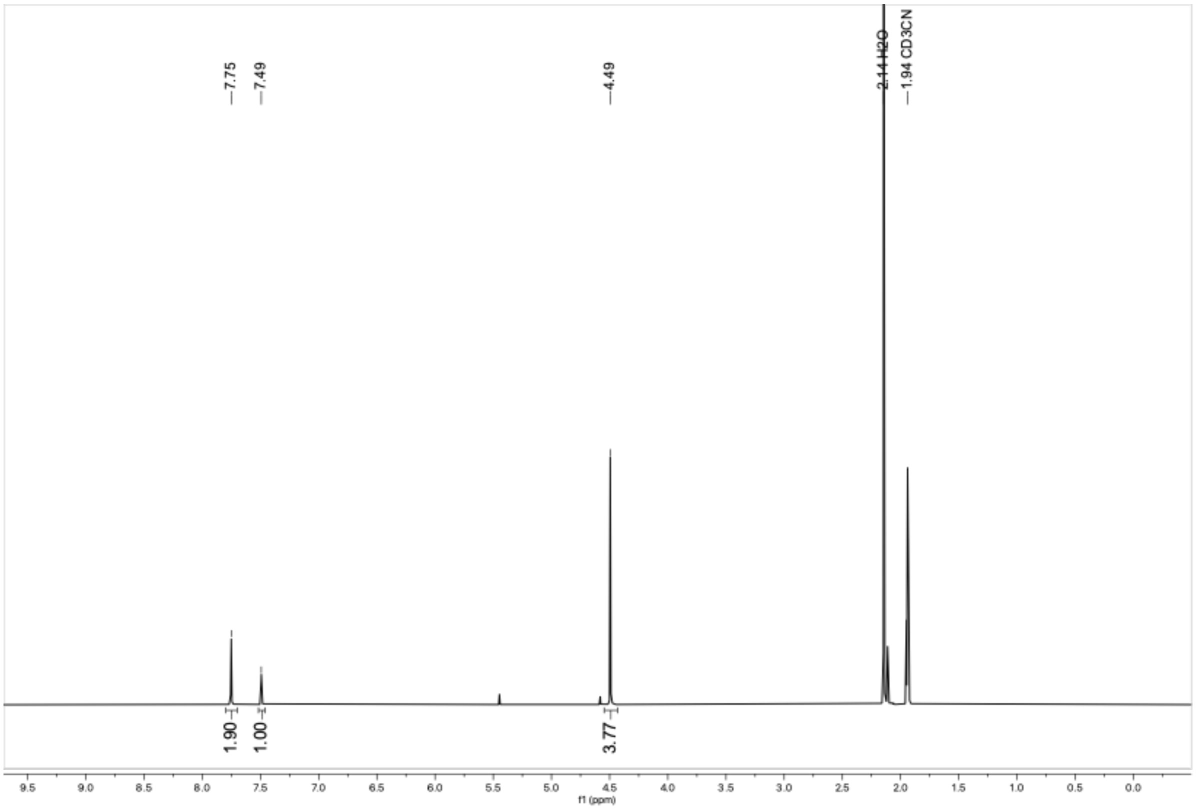


**Figure S3.** $^1$H NMR of 1,3-bis(bromomethyl)-5-iodobenzene (**1**) in $CD_3CN$ at 23 ºC.

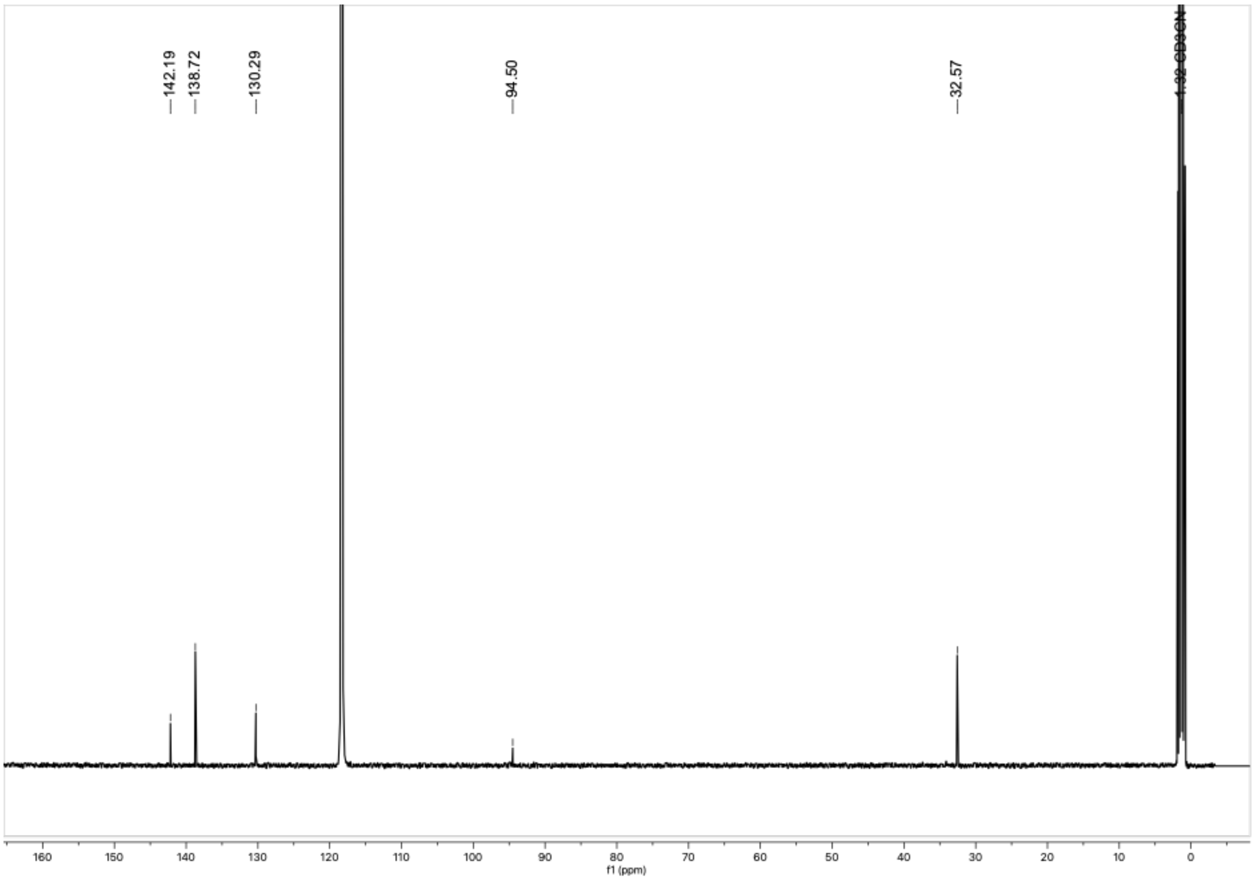


**Figure S4.** $^{13}C$ NMR of 1,3-bis(bromomethyl)-5-iodobenzene (**1**) in $CD_3CN$ at 23 ºC.

## Synthesis of Cyclic PEG (2 kDa)-Aryl Iodide (2)

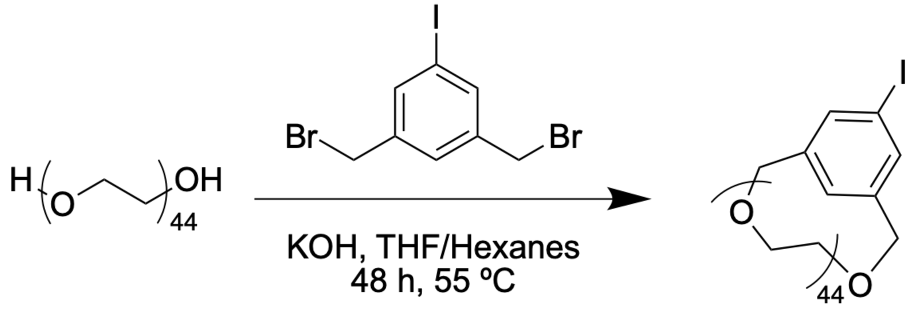


The following procedure was modified from Hadjichristidis *et al.*[4] A two-neck round bottom flask was charged with a stir bar, then finely powdered KOH (906.7 mg, 70 eq, 16.2 mmol) was added as a solid. Next, 40 mL anhydrous THF and 10 mL hexanes were added to the flask. The round bottom flask was affixed with a reflux condenser and the reaction mixture was heated to reflux (60 ºC). Separately, PEG (2 kDa) (461.8 mg, 1 eq, 230.9 μmol) and 1,3-bis(bromomethyl)-5-iodobenzene (90.0 mg, 1 eq, 230.9 μmol) were dissolved in 50 mL anhydrous THF and then added to the flask at a rate of 4.2 mL/h using a syringe pump. Then, following the completion of the precursor addition, the reaction was stirred for an additional 24 hours while refluxing (60 ºC). Next, the reaction mixture was filtered to remove KOH, then the solvents were removed under reduced pressure. The crude product was dissolved in DCM and purified by one precipitation in hexanes followed by three fractionations in toluene/hexanes as the good solvent and non-solvent, respectively. Finally, the product was purified using preparative HPLC with a 30-100% MeCN against $H_2O$ gradient (Both with 0.1% TFA additive). The product was isolated as a white solid (205.2 mg, 92.4 μmol, 40% yield).

**Physical State:** White solid
**$^1$H NMR (500 MHz, $CD_3CN$):** δ 7.64 (s, 2H), 7.30 (s, 1H), 4.48 (s, 4H), 3.70 – 3.20 (m, 179H).

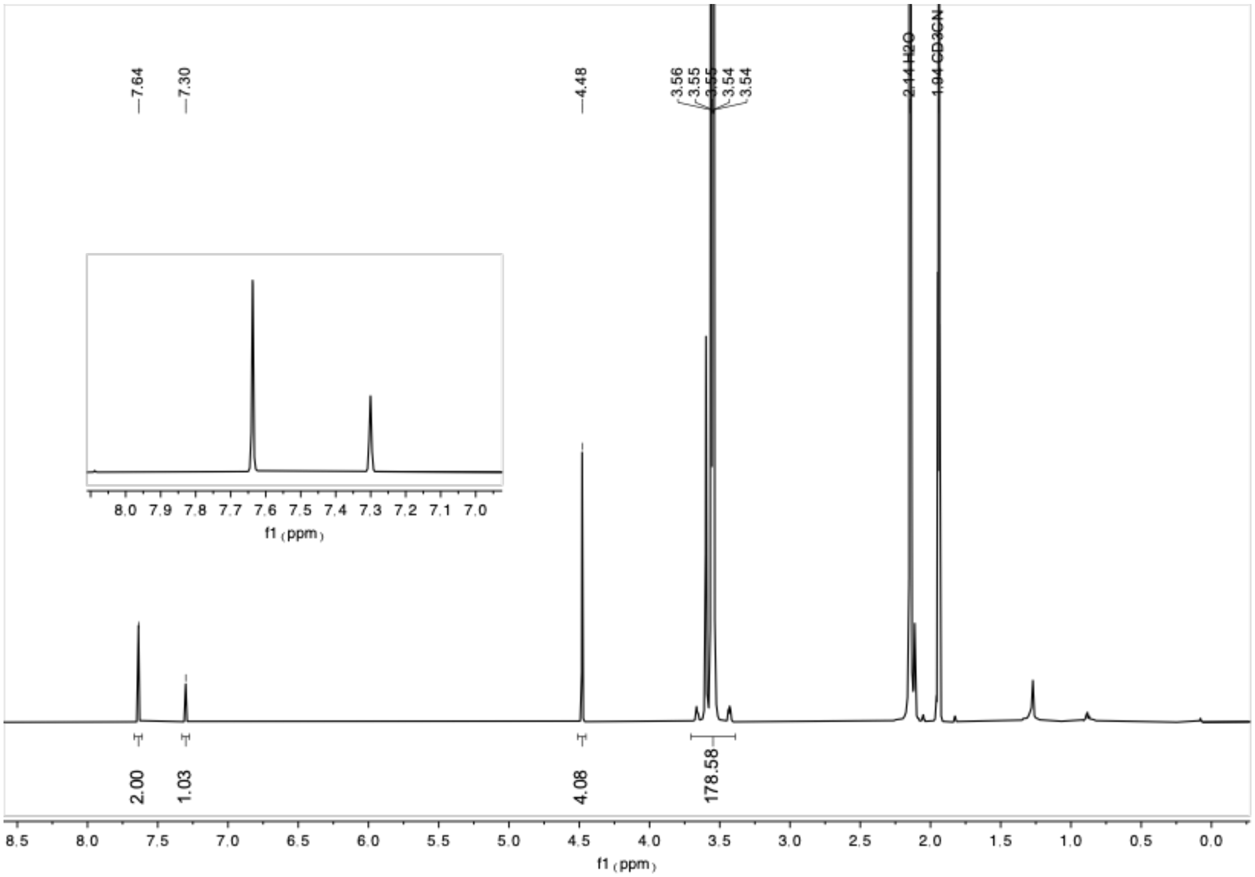


**Figure S5.** $^{1}$H NMR of cyclic PEG (2 kDa)-aryl iodide (**2**) in $CD_3CN$ at 23 ºC. Note that this data is also shown in Figure 1 of the main text, shown larger here for easier viewing.

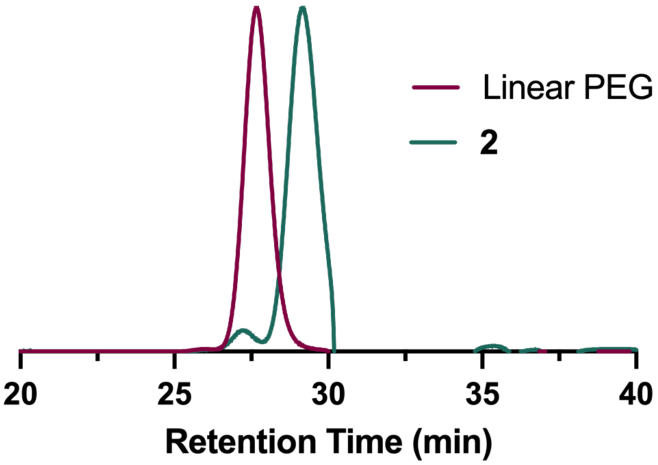


**Figure S6.** DMF SEC of cyclic PEG (2 kDa)-aryl iodide (**2**) and commercial linear PEG (2 kDa). Note that this data is also shown in Figure 1 of the main text, shown larger here for easier viewing. The SEC analysis for **2** is as follows: $M_n$ – 2.3 kDa, $M_w$ – 2.6 kDa, $Đ$ – 1.15.

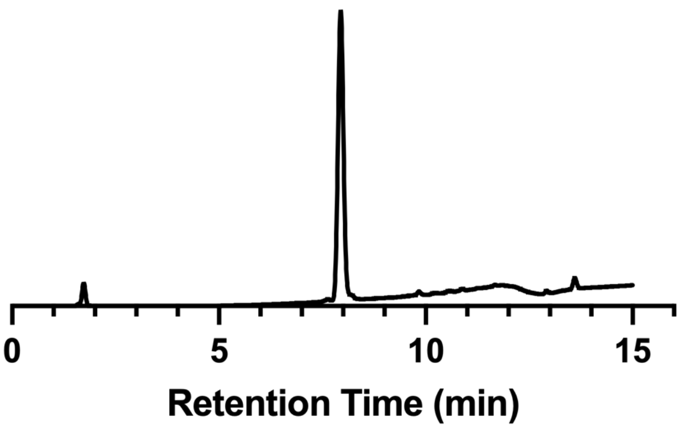


**Figure S7.** Analytical HPLC of cyclic PEG (2 kDa)-aryl iodide (**2**) at 280 nm.

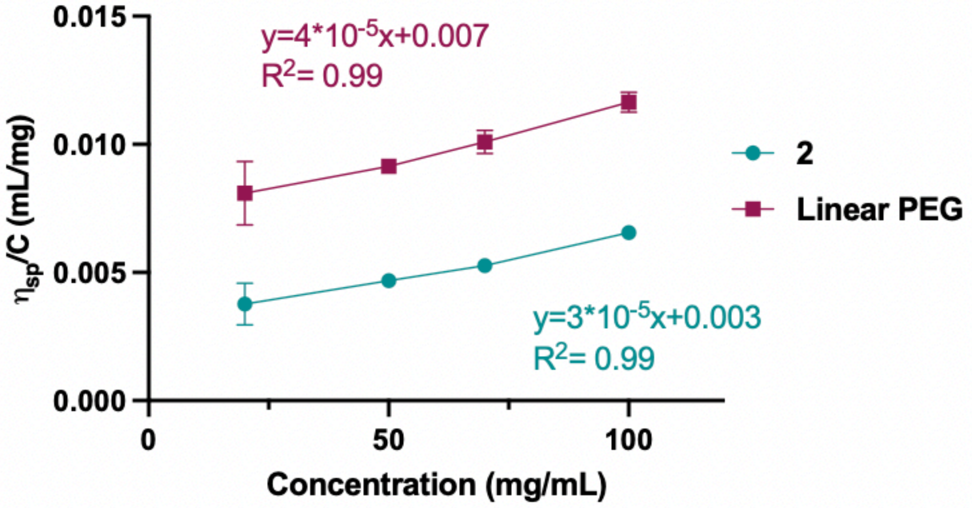


**Figure S8.** Intrinsic viscosity of cyclic PEG (2 kDa)-aryl iodide (**2**) and linear mPEG (2 kDa)-aryl iodide. The y-intercepts represent the intrinsic viscosity [η] for **2** and linear mPEG (2 kDa)-aryl iodide as 0.003 and 0.007 mL/mg, respectively.

## Synthesis of Cyclic PEG (2 kDa) [(Me-DalPhos)AuCl][$SbF_6$] (3)

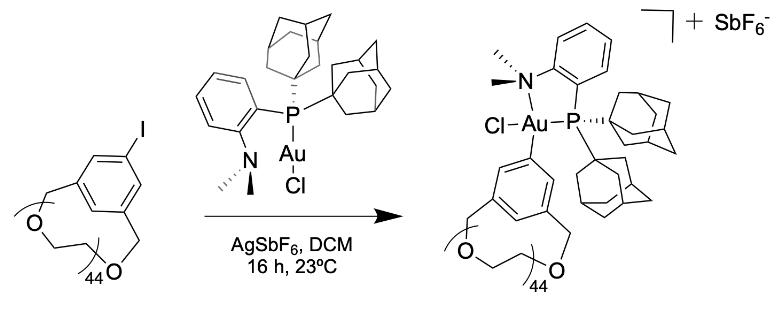


$AgSbF_6$ was removed from a dinitrogen-filled glove box prior to the reaction. (Me-DalPhos)AuCl (9.4 mg, 1.5 eq, 14.3 μmol) and $AgSbF_6$ (9.8 mg, 3 eq, 28.7 μmol) were added together with 1.5 mL DCM and stirred for 1 minute. Visible particulates crashed from the solution. Next, **3** (21.0 mg, 1 eq, 9.5 μmol) was added with an additional 500 μL DCM. The reaction was stirred for 18 hours at 23 ºC. The crude product was filtered through Celite, then the DCM was removed under reduced pressure. The product was isolated as an off-white solid (24 mg, 7.98 μmol, 84% yield). The product purity by weight was determined to be 85% by $^{1}H$ NMR.

**Physical State:** Off-white solid
**$^{1}H$ NMR (600 MHz, $CD_3CN$):** δ 8.10 – 7.94 (m, 3H), 7.89 – 7.80 (m, 1H), 7.58 (s, 2H), 7.24 – 7.14 (m, 1H), 4.52 (s, 4H), 3.72 – 3.40 (m, 177H), 1.82 – 1.63 (m, 26H).
**$^{31}P\{^{1}H\}$ NMR (243 MHz, $CD_3CN$):** δ 75.25, 52.22.

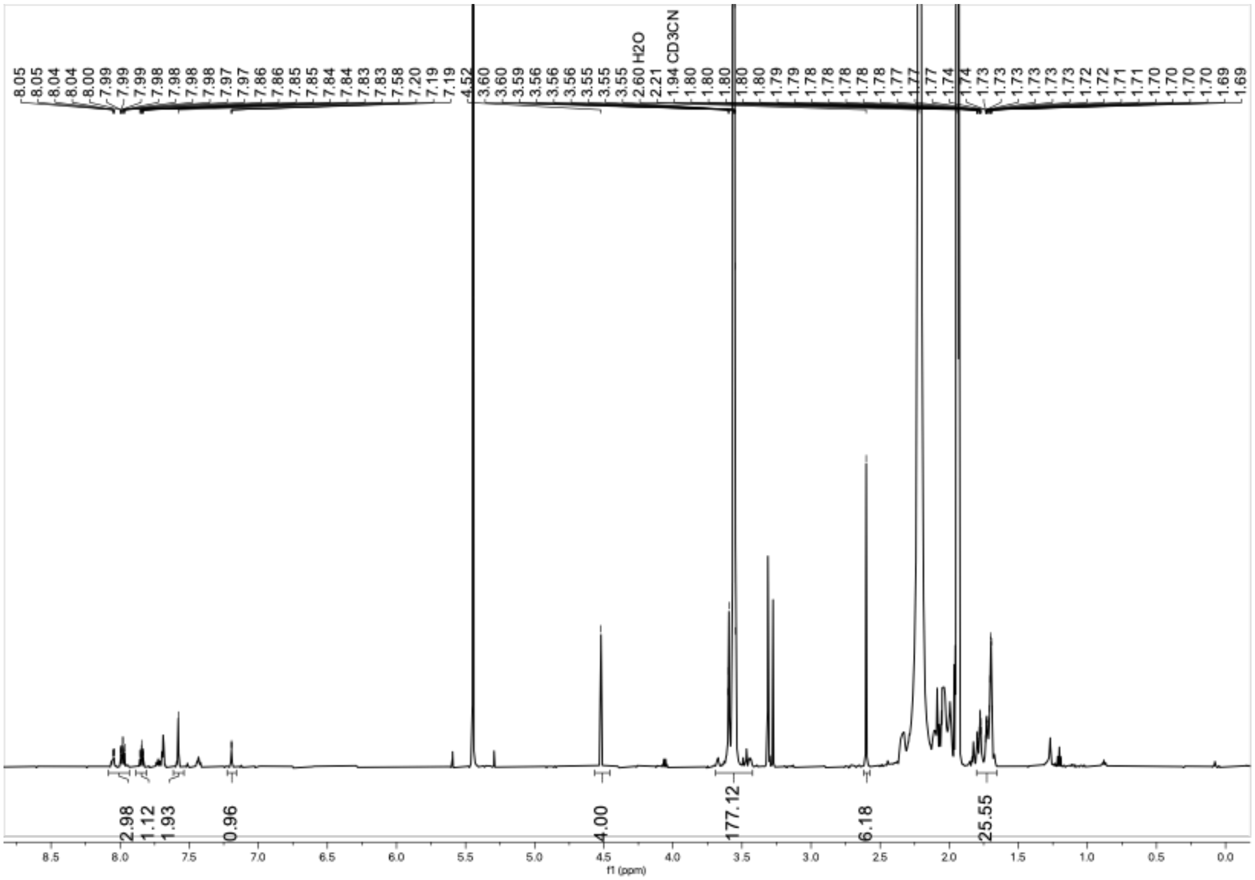


**Figure S9.** $^{1}$H NMR of cyclic PEG (2 kDa) [(Me-DalPhos)AuCl][SbF$_6$] (**3**) in $CD_3CN$ at 23 ºC.

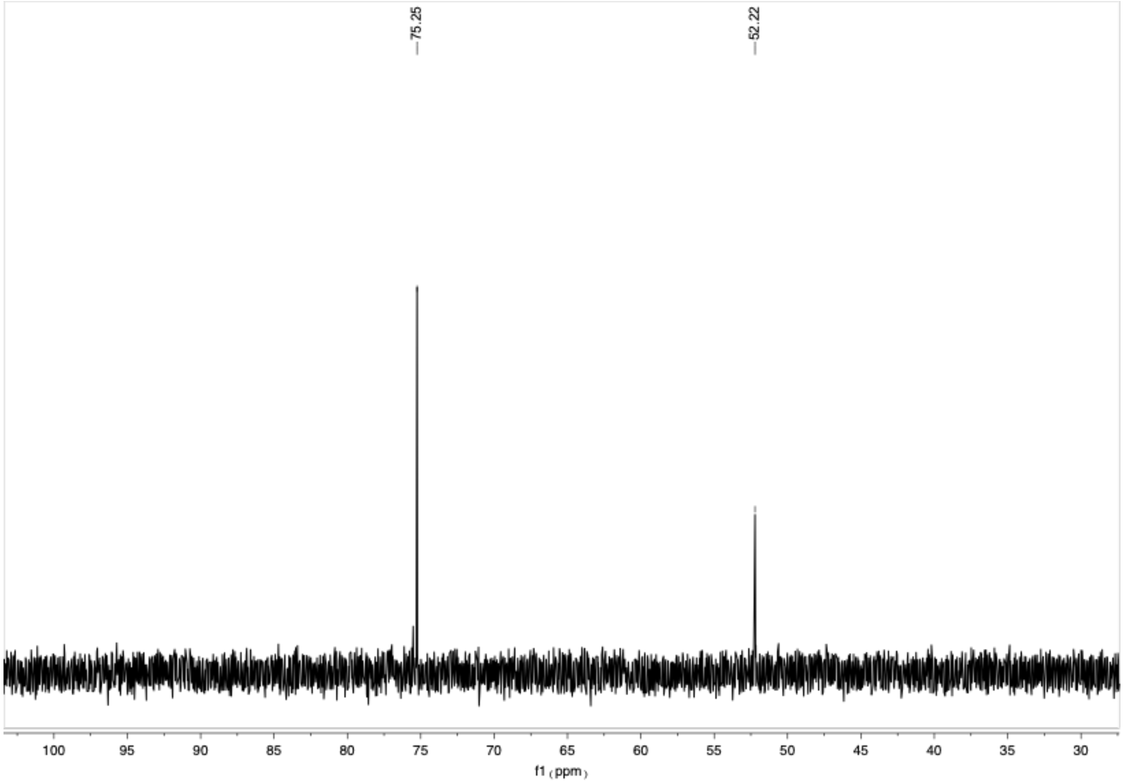


**Figure S10.** $^{31}P\{^{1}H\}$ NMR of cyclic PEG (2 kDa) [(Me-DalPhos)AuCl][$SbF_6$] (**3**) in $CD_3CN$ at 23 ºC. The desired Au(III) resonance occurs at 75 ppm. The resonance at 52 ppm corresponds to residual (Me-DalPhos)Au(I)Cl starting material. Note that this data is also shown in Figure 1 of the main text, shown larger here for easier viewing.

## Synthesis of Linear PEG (2 kDa) [(Me-DalPhos)AuCl][$SbF_6$] (4)

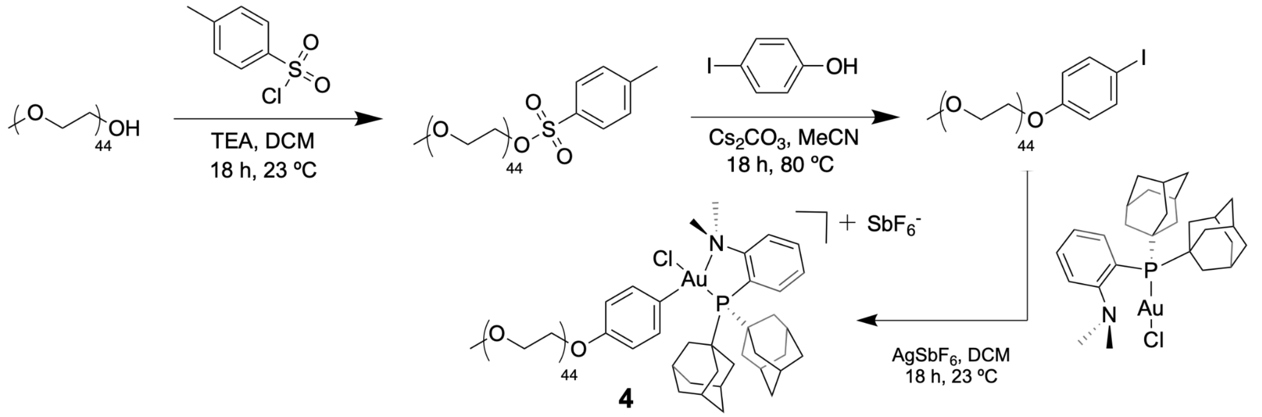


**Scheme 2.** Synthesis of linear mPEG (2 kDa)[(Me-DalPhos)AuCl][$SbF_6$] (**4**).

Linear mPEG (2 kDa) Au(III) reagent was synthesized as reported previously.[4] Briefly, commercial mPEG-OH (2 kDa) was tosylated under basic conditions at 23 ºC. Next, the resulting mPEG-tosyl was arylated with 4-iodophenol under refluxing conditions. Finally, the mPEG-aryl iodide underwent oxidative addition with (Me-DalPhos)AuCl to produce linear mPEG (2 kDa) [(Me-DalPhos)AuCl][$SbF_6$] (**4**). All spectra match those of literature values.

## T4 Lysozyme V131C (T4L) Protein Expression

T4 lysozyme V131C (T4L) protein expression and purification was adapted from literature procedures.[5] T4 lysozyme V131C (T4L) Sequence (Calculated Mass: 18605.36 Da): MNIFEMLRIDEGLRLKIYKDTEGYYTIGIGHLLTKSPSLNAAKSELDKAIGRNTNGVITKD EAEKLFNQDVDAAVRGILRNAKLKPVYDSLDAVRRAALINMVFQMGETGVAGFTNSLR MLQQKRWDEAACNLAKSRWYNQTPNRAKRVITTFRTGTWDAYKNL.

*E. coli* host BL21(DE3) (Invitrogen) was used to express T4 lysozyme V131C (T4L) using an ampicillin resistant expression vector obtained from Prof. Wayne Hubbell (UCLA). To each of two 2 L flasks each containing 750 mL of previously autoclaved LB Broth (Miller) with ampicillin (50 µg/mL) was added 5 mL of a saturated 18 hour culture inoculated from a glycerol stock. The culture was grown at 37 °C with 250 rpm shaking for ca. 6 hours before the OD600 reached ~0.75 and the culture was induced with 1 mM IPTG. The culture continued to shake at 37 °C at 250 rpm for approximately 2 hours. The cultures were harvested by centrifugation at 4000 rpm for 15 min to yield a cell pellet. The pellet was resuspended in 20 mL lysis buffer (25 mM Tris, 25 mM MOPS, 0.2 mM EDTA, pH 7.6) by stirring vigorously. Spontaneous bacterial lysis occurred during resuspension as evident by increased viscosity of the suspension due to DNA release. Bacterial lysis was likely due to proteolytic activity of the lysozyme present in the bacteria. One subsequent freeze-thaw cycle ensured complete lysis of the bacteria. Benzoase (0.2 µL/mL) was then added to the solution and incubated at 25 °C for 15 min. Bacterial debris was then separated by centrifugation at 17,000 rpm for 25 min at 4 °C. The supernatant was loaded onto a 5 mL gravity SP Sepharose Fast Flow column (Cytiva) and washed with 200 mL lysis buffer. The column was then washed with two column volumes of lysis buffer with increasing NaCl content (gradient from 0 M to 0.5 M in 0.1 M steps) to elute the desired protein. SDS-PAGE was run on all fractions and under reducing conditions with Coomassie Blue staining. Pure fractions were combined and solvent exchanged into storage buffer (20 mM PBS, pH 6.5) and concentrated to ~15 mL using an Amicon 3K Ultra-15 Centrifugal Filter (Millipore). Protein was further purified by preparative SEC-FPLC using an isocratic method in 20 mM PBS, pH 6.5. Pure fractions were concentrated and stored in storage buffer as described above. The purified protein was analyzed by LCMS and SDS-PAGE confirming sample purity and molecular weight (main text Figure 2). Concentration was determined by A280 (Extinction coefficient = 25,440 $M^{-1}$ $cm^{-1}$) The protein sample was diluted with storage buffer to 76 µM and aliquots were flash frozen and stored in a -80 °C freezer.

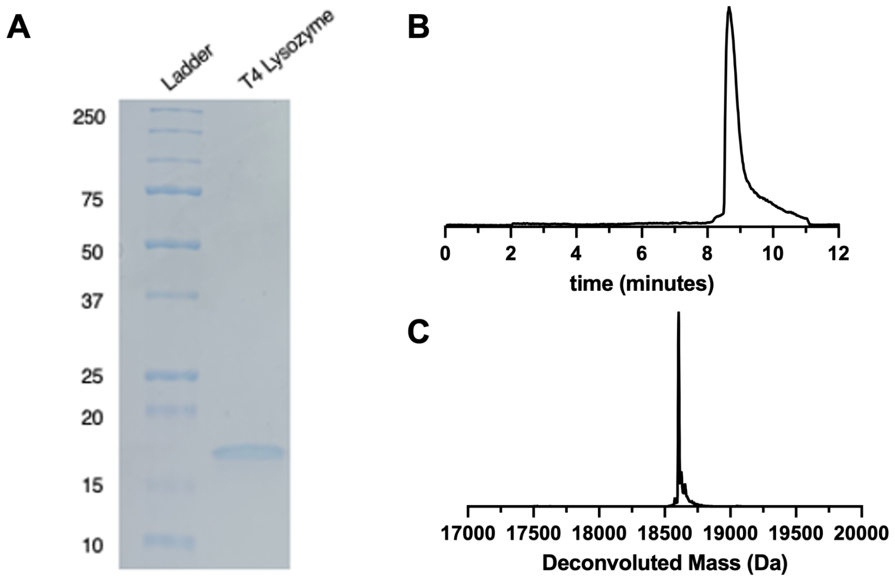


**Figure S11.** A) SDS-PAGE gel of T4 lysozyme following purification. B) LCMS total ion chromatogram (TIC) of T4 lysozyme following purification. C) LCMS deconvoluted mass of T4 lysozyme following purification. Expected mass: 15,866.84 Da. Observed mass: 15,866.23 Da.

# Preparation of T4L-PEG Conjugates

## General Experimental Information

Protein-polymer conjugates were purified by FPLC on a Bio-Rad BioLogic DuoFlow chromatography system. All purifications were carried out at 4 °C. All buffers were freshly prepared and filtered over a Thermo Scientific Nalgene 565-0020 Filter Unit, 0.2 µm PES prior to use. Size exclusion chromatography (SEC) purifications were performed using a Superdex 75 Increase 10/300 GL column. All protein purifications were monitored at wavelengths of 254 nm and 280 nm. A standard isocratic method was used for T4L: 20 mM PBS, pH 6.5 over 37 minutes.

Protein concentration measurements were determined on a NanoDrop 2000 UV-Vis spectrophotometer at 280 nm. The extinction coefficient of T4L was calculated by ProtoParam on ExPASy based on the amino acid sequence of the protein ($\varepsilon$ = 25,440 $M^{-1}cm^{-1}$).

Sodium dodecyl sulfate-polyacrylamide gel electrophoresis (SDS-PAGE) was carried out in a Mini-PROTEAN Tetra Cell system (Bio-Rad) connected to a PowerPac HC (BioRad) power supply using Bio-Rad Any kD™ Mini-PROTEAN® TGX™ Precast Gels at 195 V and 3 A for 30 minutes in a running buffer (25 mM Tris, 192 mM Glycine, 0.1% (w/v) SDS, pH 8.3). Precision Plus ProteinTM Dual Xtra Prestained Protein Standards (2 µL) were used as protein ladder in all SDS-PAGE analysis. Laemmli 2x Concentrate (Sigma) containing 4% SDS, 20% glycerol, 0.004% bromophenol blue, and 0.125 M Tris HCl at a pH of ca. 6.8 was used to load all protein and conjugate samples. Protein bands were visualized by staining the gels in an aqueous solution (0.1% Coomassie Brilliant Blue R 250, 45% MeOH, 9% acetic acid) and microwaving for 30 seconds followed by agitation for 15 minutes. Destaining was carried out by submerging the gels in an aqueous destaining solution (10% MeOH, 14% acetic acid), microwaving for 30 seconds, and agitating for several hours until the background of the gel became fully destained.

LCMS analysis was carried out using an Agilent 6530 ESI-Q-TOF. Protein analyses were carried out using an Agilent ZORBAX 300SB C3 column (3.5 µm, 3.0 × 150 mm). Liquid chromatography method used for T4L and its conjugates: column temperature: 40 °C, flow rate: 0.6 mL/min, gradient: 90% water (0.1% formic acid (FA)) for 2 minutes; 90%-9% water (0.1% FA) 2-11 minutes; 5% water (0.1% FA) from 11-14 min with acetonitrile as the co-solvent.

## T4L-PEG Conjugation Procedure: General Conditions

General conditions for reductive eliminations are as follows: 70 µM T4L, PBS pH 6.5, 4 equiv. TCEP·HCl, 3 equiv. Au(III), 18 hours, 37 °C. To a 50 µL solution of T4L at 76 µM, 5 µL of a 3 mM TCEP·HCl (4 eq) solution in PBS was added. The protein underwent disulfide reduction for 1 hour at 37 ºC. Next, 5 µL of either **3** or **4** was added to the reaction mixture. Stock solutions of **3** and **4** were prepared in MeCN prior to the procedure (6.2 mg/mL and 5.3 mg/mL, respectively) such that 3 eq of each oxidative addition complex were added. The percent weight purity of **3** was taken into account. After 18 hours, the reaction was stopped by dilution in SDS-PAGE conditions (See below) to produce T4L-cyclic PEG (**5**) and T4L-linear PEG (**6**) conjugates.

*General SDS-PAGE Sample Preparation Procedure:* 1 µL of a given reaction mixture was added to 19 µL of Laemmli loading buffer. For samples run under reducing conditions, the Laemmli loading buffer was prepared to contain 5% mercaptoethanol by volume and samples were heated at 90 ºC for five minutes prior to loading. Samples were loaded onto SDS-PAGE gel and run as described above in the general experimental information.

## T4L Reduction TCEP·HCl Equivalent Screen

To a 50 µL solution of T4L at 76 µM, 5 µL of a TCEP·HCl solution in PBS was added. The TCEP·HCl stock solution concentration was varied between 3, 4.5, and 6 mM (4, 6, and 8 equivalents, respectively). The protein underwent disulfide reduction for 1 hour at either 23 ºC or 37 ºC, then the samples underwent the general SDS-PAGE sample preparation procedure in non-reducing Laemmli loading buffer as described above.

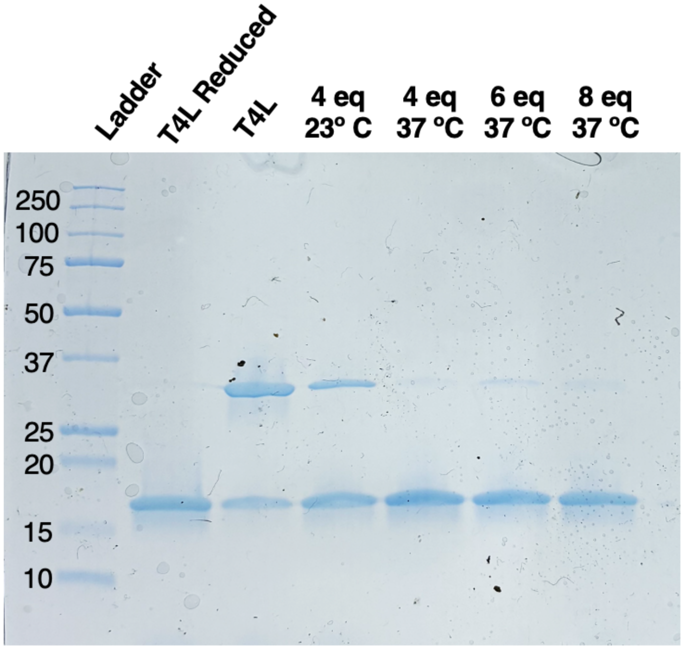


**Figure S12.** SDS-PAGE of T4 lysozyme (T4L) in PBS buffer (pH 6.5) reduced with 4, 6, or 8 eq TCEP·HCl for 1 hour at either 23 ºC or 37 ºC. These samples were run in conventional Laemmli buffer (non-reducing).

## PEG Equivalent and Temperature Screen

To a 50 µL solution of T4L at 76 µM, 5 µL of a 3 mM TCEP·HCl (4 eq) solution in PBS was added. The protein underwent disulfide reduction for 1 hour at 37 ºC. Next, 5 µL of **3** or **4** were added to the solution at either 1.3 eq or 3 eq, with all PEG stock solutions prepared in MeCN. The reactions were allowed to proceed at either 23 ºC or 37 ºC for 3 hours. Then, the samples underwent the general SDS-PAGE sample preparation procedure as described above.

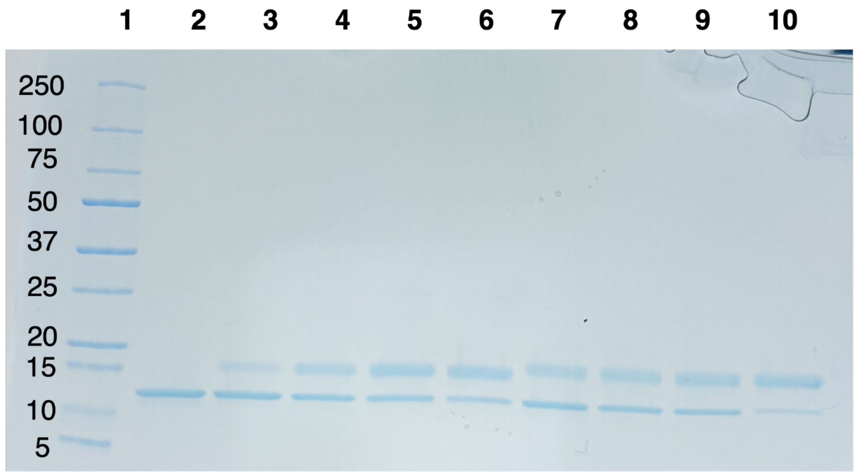


| Lane | Condition |
|---|---|
| 1 | Ladder |
| 2 | T4L-reduced |
| 3 | Cyclic PEG (**3**,1.3 eq), 23 ºC |
| 4 | Cyclic PEG (**3**,1.3 eq), 37 ºC |
| 5 | Cyclic PEG (**3**, 3 eq), 23 ºC |
| 6 | Cyclic PEG (**3**, 3 eq), 37 ºC |
| 7 | Linear mPEG (**4**, 1.3 eq), 23 ºC |
| 8 | Linear mPEG (**4**, 1.3 eq), 37 ºC |
| 9 | Linear mPEG (**4**, 3 eq), 23 ºC |
| 10 | Linear mPEG (**4**, 3 eq), 37 ºC |

All reductive eliminations occurred for 3 hours

**Figure S13.** SDS-PAGE of crude T4L-PEG conjugates synthesized in PBS buffer (pH 6.5) for 3 hours, utilizing either 1.3 or 3 equivalents of **3** and **4** at either 23 ºC or 37 ºC. These samples were run in a reducing Laemmli buffer.

## Extended Time Screen

To a 50 µL solution of T4L at 76 µM, 5 µL of a 3 mM TCEP·HCl (4 eq) solution in PBS was added. The protein underwent disulfide reduction for 1 hour at 37 ºC. Next, 5 µL of **3** or **4** were added to the solution at either 1.3 eq or 3 eq, with all PEG stock solutions prepared in MeCN. The reactions were allowed to proceed at either 23 ºC or 37 ºC for 18 hours, then the samples underwent the general SDS-PAGE sample preparation procedure as described above.

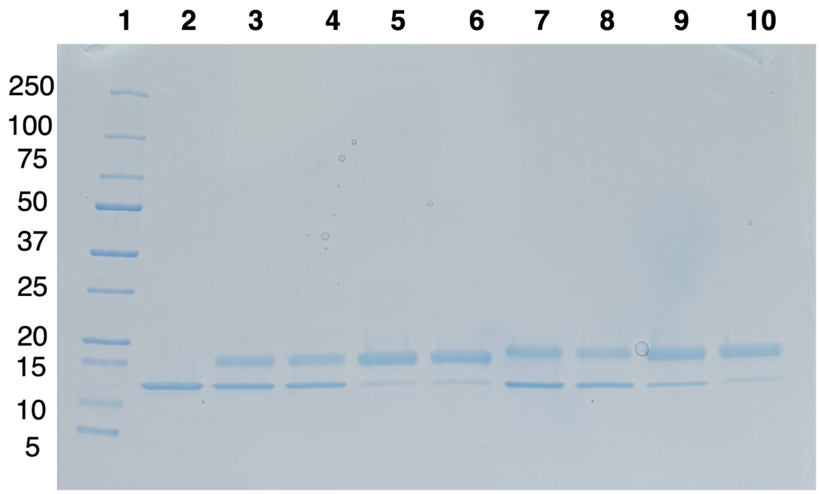


| Lane | Condition |
|---|---|
| 1 | Ladder |
| 2 | T4L-reduced |
| 3 | Cyclic PEG (**3**,1.3 eq), 23 ºC |
| 4 | Cyclic PEG (**3**,1.3 eq), 37 ºC |
| 5 | Cyclic PEG (**3**, 3 eq), 23 ºC |
| 6 | Cyclic PEG (**3**, 3 eq), 37 ºC |
| 7 | Linear mPEG (**4**, 1.3 eq), 23 ºC |
| 8 | Linear mPEG (**4**, 1.3 eq), 37 ºC |
| 9 | Linear mPEG (**4**, 3 eq), 23 ºC |
| 10 | Linear mPEG (**4**, 3 eq), 37 ºC |

All reductive eliminations occurred for 18 hours

**Figure S14.** SDS-PAGE of crude T4L-PEG conjugates synthesized in PBS buffer (pH 6.5) for 18 hours, utilizing 1.3 or 3 equivalents of **3** and **4** at either 23 ºC or 37 ºC. These samples were run in a reducing Laemmli buffer.

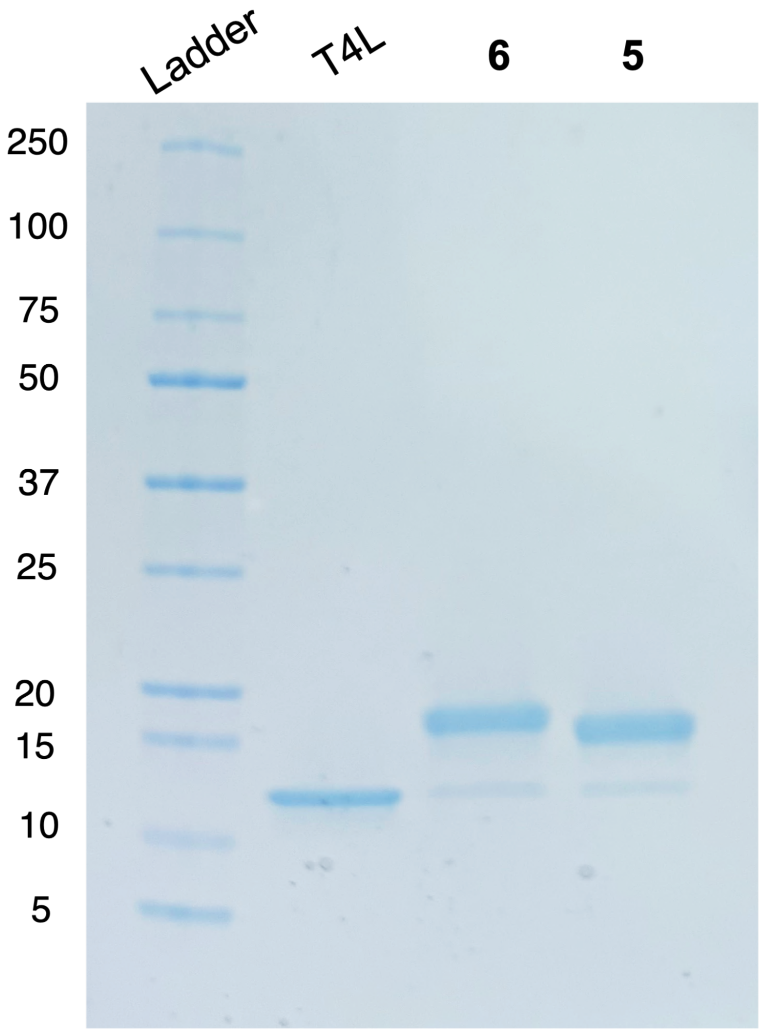


**Figure S15.** SDS-PAGE of crude T4L-PEG conjugates **6** and **5** synthesized in PBS buffer (pH 6.5) for 18 hours at 23 ºC, utilizing 3 equivalents of **4** and **3**, respectively. These samples were run in a reducing Laemmli buffer. Based on ImageJ optical densitometry, conversion to conjugate **6** is 80% and conversion to **5** is 84%.

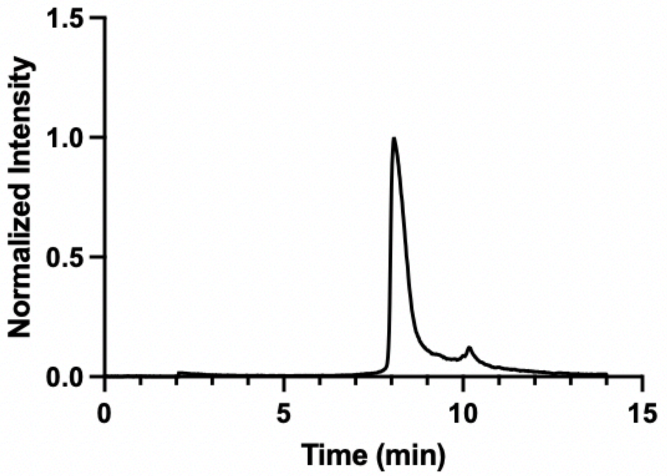


**Figure S16.** LCMS total ion chromatogram (TIC) of T4L-cyclic PEG (**5**) following purification. The major peak at 8 min corresponds to the deconvoluted mass spectra shown in Figure 2C of the main text. The minor peak at 10 min corresponds to 2 kDa PEG.

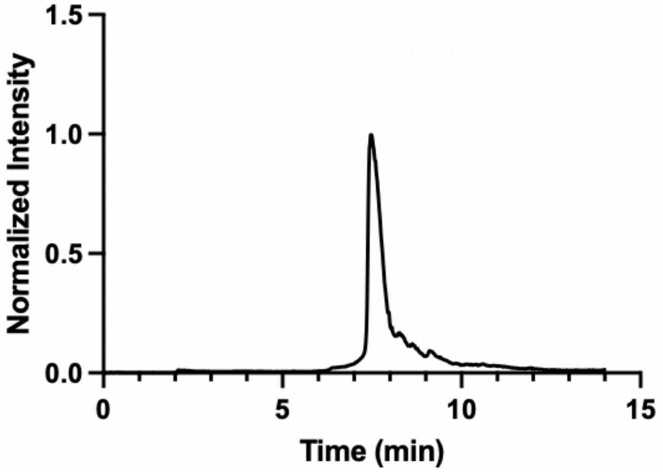


**Figure S17**. LCMS total ion chromatogram (TIC) of T4L-linear PEG (**6**) following purification. The major peak at 7.5 min corresponds to the deconvoluted mass spectra shown in Figure 2D of the main text.

## Characterization of PEGylated-T4 Lysozyme Conjugates

## Circular Dichroism (CD) and Stability Assessment

T4L and purified **5** and **6** were diluted to 7 µM in 300 µL total volume of PBS buffer (pH 6.5). CD spectra were taken ranging from 200-280 nm at 25 °C. Thermal denaturation analysis was performed by calculating the relative helicity ((mdeg-$mdeg_{min}$)/$mdeg_{max}$) at 223 nm from 20 – 100 °C.

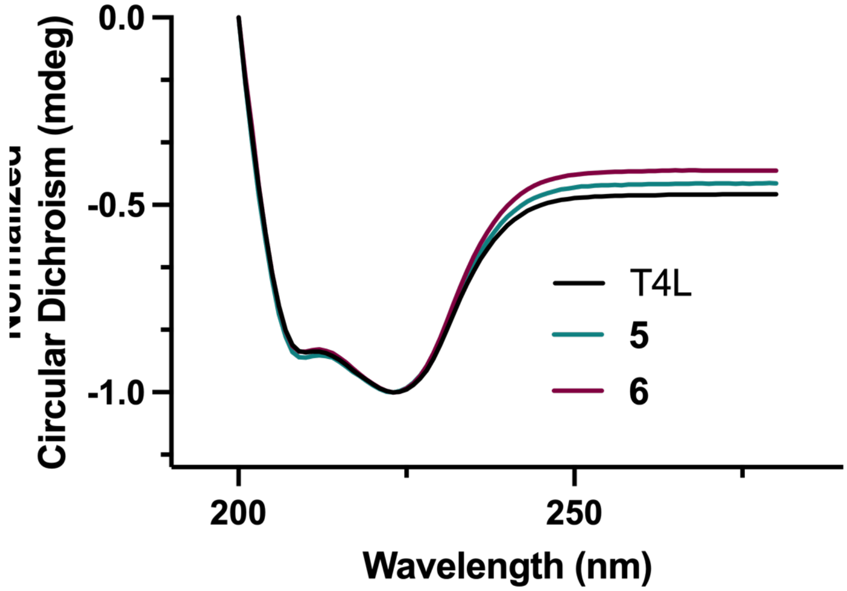


**Figure S18.** CD spectrum of T4L and T4L conjugates (**5** and **6**) at 25 ºC showing no significant difference in helicity. Data has been normalized to the global minimum for each sample. Local minima for each sample are as follows – T4L: 210 and 223 nm, **5**: 209 and 223 nm, **6**: 210 and 223 nm. Note that this data is also shown in Figure 3 of the main text, shown larger here for easier viewing.

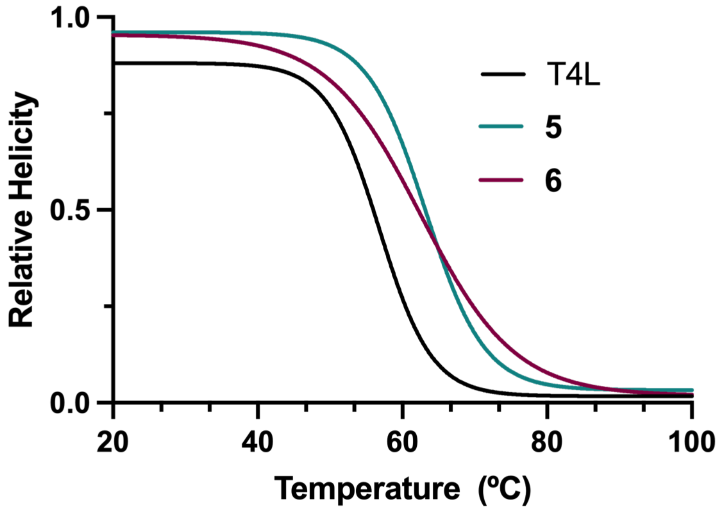


**Figure S19.** CD thermal denaturation curves at 223 nm of T4L and T4L conjugates (**5** and **6**) showing no significant difference in melting temperature ($T_m$) between 20 – 100 °C. Specifically, $T_m$ values for each sample are as follows – T4L: 56.8 ºC, **5**: 63.2 ºC, **6**: 62.6 ºC.

# Activity Assay

The lysozyme activity assay was conducted using the Invitrogen™ ENZChek™ lysozyme assay kit with minor modifications to the manufacturer instructions. Specifically, 50 µL of T4L, **5**, and **6** at 7 µM were pipetted into a 96 well plate. Sample concentration was determined *via* A280 (Extinction coefficient = 25,440 $M^{-1}$ $cm^{-1}$). For each sample, three replicates were performed. Then, 20 µL of a 5% solution fluorescein-labeled *Micrococcus luteus* in PBS (pH 6.5) was added to each well. The plate was incubated at 37 ºC for 30 minutes. Fluorescein fluorescence from cell lysis was measured (Excitation 490 nm, emission 530 nm). All activity values are reported relative to fresh unmodified T4L activity.

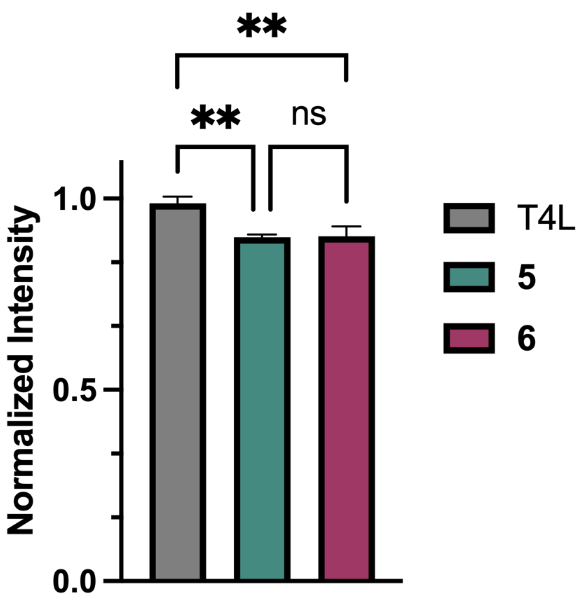


**Figure S20.** Lysozyme activity assay fluorescence output of T4L, **5**, and **6**. N = 3 for each group. Ordinary one-way ANOVA statistical analysis was performed. **p < 0.005. ns = not significantly different. Note that this data is also shown in Figure 4 of the main text, shown larger here for easier viewing.

# ICP-OES Analysis

Measurements were performed on an Agilent 5110 ICP-OES (Inductively coupled plasma-optical emission spectrometer). A Sigma-Aldrich 1000 ppm (Lot value: 999 ppm ± 2 ppm, 5% w/w HCl) Gold Standard for ICP was used as a stock solution to create standards of concentrations 50 ppb, 100 ppb, and 1000 ppb. Solutions were prepared using volumetric flasks, volumetric pipettes, and an Eppendorf pipette for aliquoting of the Au stock solution. A calibration curve was generated for each standard by integrating the signal corresponding to the characteristic Au emission (242.79 nm). A Yttrium internal standard (2 ppm in 2% $HNO_3$) was run simultaneously with all samples and the characteristic Y emission was measured at 371.03 nm.

Samples **5** and **6** were each quantitatively transferred with multiple washes of MilliQ water to a 14 mL Falcon tube, acidified by the addition of 0.2 mL $HNO_3$ (FisherChemical Trace Metal Grade Nitric Acid; certified [Au] < 0.1 ppb) and diluted to a total volume of 4.0 mL with MilliQ water. This solution was sonicated for 5 minutes and then immediately analyzed. No Au was detected within the range of the calibration curve, indicating [Au] < 50 ppb in each sample, which is near the lower detection limit for this instrument.

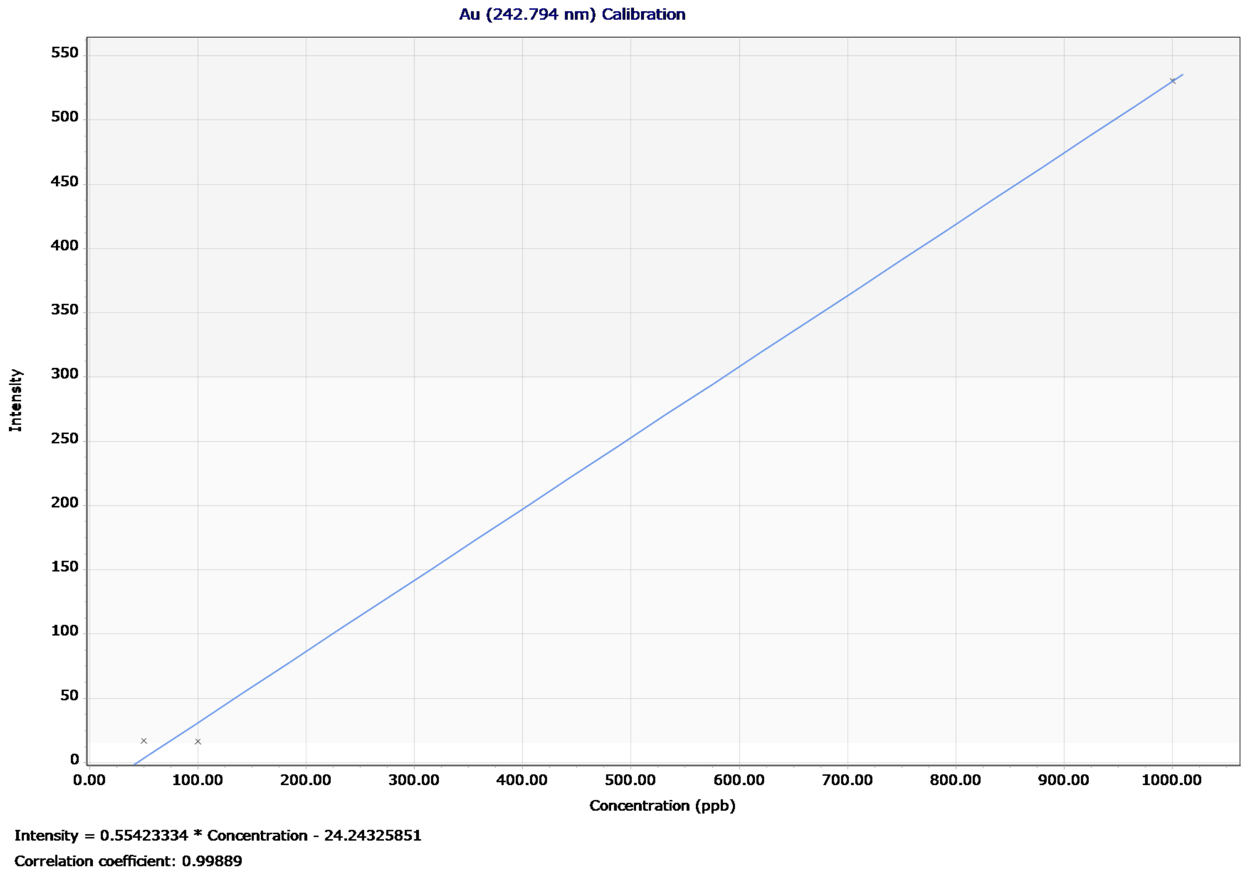


**Figure S21**. ICP-OES Au calibration curve.

# Molecular Dynamics Simulations

Molecular dynamics (MD) simulations were performed using Amber 22 program[6] and AmberTools 23 packages.[7] For the MD simulations, the protein structure from x-ray diffraction was used for the simulations (PDB code: 2HUK).[8] Hydrogen atoms were added using Protein Plus and Protoss online server.[9,10] MD simulations in explicit water were performed using the GPU accelerated code (pmemd).[11,12] For the protein scaffold, Amber 19 force field (ff19SB) parameters were applied.[13] OPC4 parameters were assigned to water molecules.[14] The partial charges of the C131 and PEG polymer were set to fit the electrostatic potential generated at the HF/6-31G(d)[15,16] level of theory by the RESP (restrained electrostatic potential) method using models systems (Figure S16).[17] The charges were calculated according to the Merz-Singh-Kollman scheme[18,19] using Gaussian 16 program package.[20] Long-range electrostatic effects were modeled using the particle mesh Ewald method with periodic boundary conditions.[21] An 8 Å cutoff was applied to Lennard-Jones and electrostatic interactions. The polymer chain was manually attached to C131 residue. To relax the polymer conformation, the system was first minimized with a maximum cycle of 10000 under GBSA implicit solvent model[22] with positional restraints (500 kcal mol$^{-1}$ Å$^{-2}$) applied to peptide backbone atoms (C, C$_{\alpha}$, N), and then heated from 0 K to 300 K within 2 ns in an NVT ensemble with positional restraints (30 kcal mol$^{-1}$ Å$^{-2}$) on all peptide backbone atoms (C, C$_{\alpha}$, N). The system was then placed in a pre-equilibrated octahedral box with a 10 Å buffer of OPC4 water molecules using the tleap module. The systems were neutralized by addition of explicit counter ions ($Cl^{-}$). Molecular dynamics simulations were performed according to the following steps. (1) Each system was minimized over 5,000 steps with positional restraints (500 kcal mol$^{-1}$ Å$^{-2}$) applied to all atoms except water molecules and chloride ions, followed by an energy minimization over 5,000 steps with positional restraints (2.0 kcal mol$^{-1}$ Å$^{-2}$) on all peptide backbone atoms (C, C$_{\alpha}$, N). (2) The positional restraint of the ligand was removed. A 300 ps heating process was performed with periodic boundary for constant volume (NVT) with SHAKE algorithm turned on. Positional restraints (30 kcal mol$^{-1}$ Å$^{-2}$) were applied on all peptide backbone atoms (C, C$_{\alpha}$, N). (3) A 2 ns equilibrium process was performed with periodic boundary for constant pressure (NPT) and with a constant temperature of 300 K with high positional restraints (30 kcal mol$^{-1}$ Å$^{-2}$) on all peptide backbone atoms (C, C$_{\alpha}$, N). (4) A 2 ns equilibrium process was performed with constant pressure (NPT) and with a constant temperature of 300 K with low positional restraints (0.5 kcal mol$^{-1}$ Å$^{-2}$) on all peptide backbone atoms (C, C$_{\alpha}$, N). (5) To further relax the polymer, we then annealed the system by heating it from 300 K to 400 K, and then cooling it back to 300 K in 400 ps. The annealing cycle was repeated 10 times. Positional restraints (30 kcal mol$^{-1}$ Å$^{-2}$) were applied on all peptide backbone atoms (C, C$_{\alpha}$, N). (5) Three independent 1000 ns production runs were performed applying the standard simulation condition for constant pressure (NPT) and with a constant temperature of 300 K. Structures were visualized using ChimeraX[23] and VMD.[24]

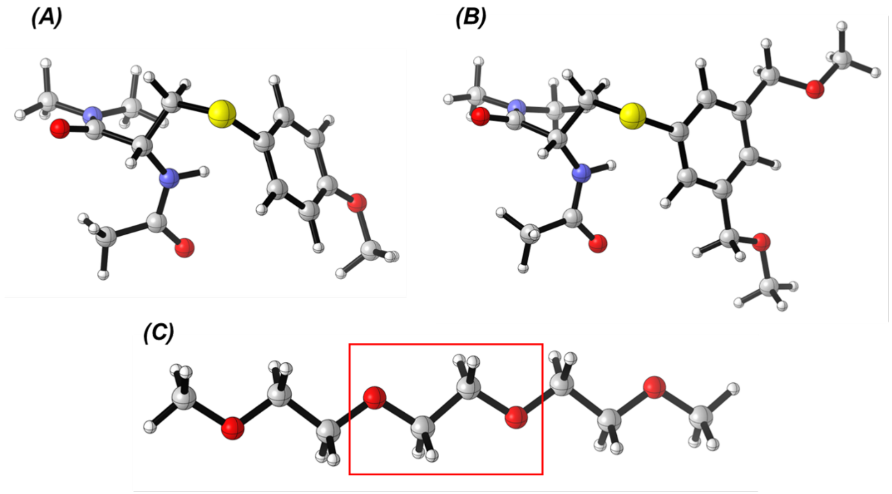


**Figure S22**. Model systems used for RESP charge calculations. (A) Model system for C131 and phenyl linker in simulations for **6**. (B) Model system for C131 and phenyl linker in simulations for **5**. (C) Model system for PEG polymer. The RESP charges of the two units in the middle (shown in red box) were average and assigned to all the PEG units in the system. All the structures are optimized with B3LYP-D3/6-31G(d).

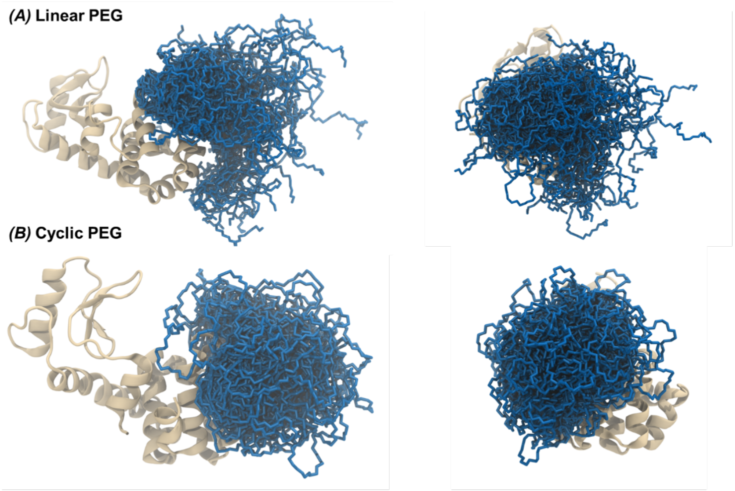


**Figure S23.** Overlay of the trajectories. (A) Trajectories for **6**. (B) Trajectories for **5**. Left: front view, right: side view. Linear PEG polymer demonstrates more conformational flexibility in MD simulations compared to cyclic PEG conjugate.

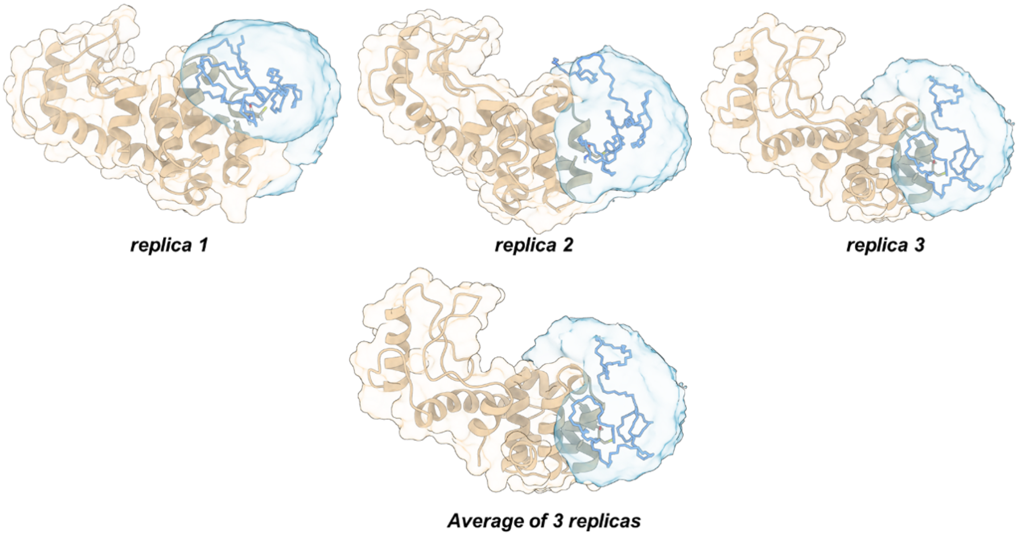


**Figure S24.** Spatial distribution functions of linear PEG (Shown in light blue isosurface) and representative structures of simulations for **6**.

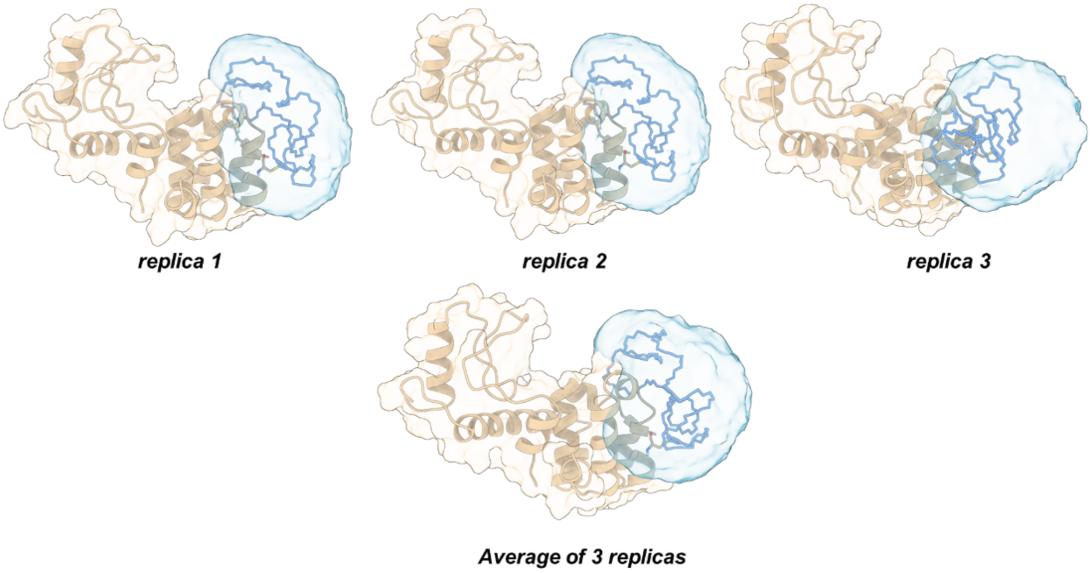


**Figure S25.** Spatial distribution functions of cyclic PEG (Shown in light blue isosurface) and representative structures of simulations for **5**.

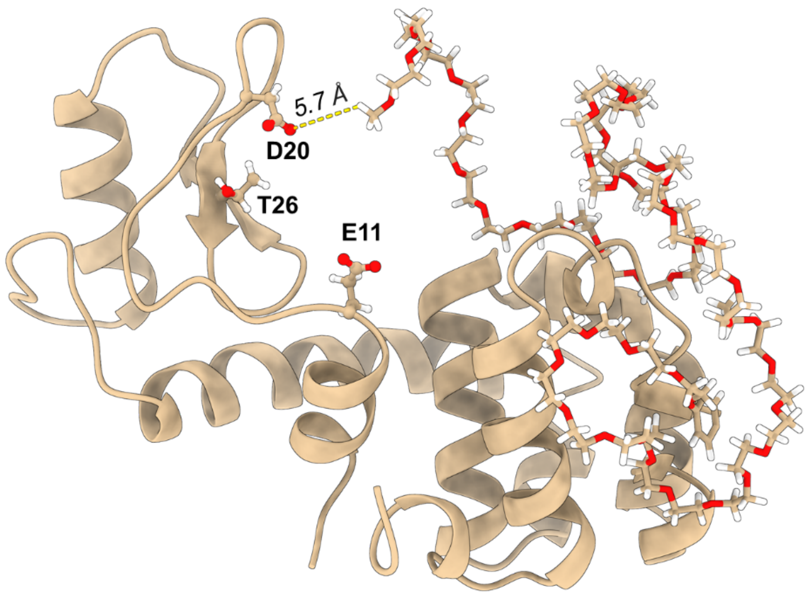


**Figure S26.** Illustration of the minimum distance studied in Figure S24 and S25. The minimum distance is defined as the smallest distance between any pair of atoms from the active site and PEG polymer. As the picture shows, the minimum distance for this structure is the distance between the carboxylic oxygen in D20 and a hydrogen in PEG (labeled by yellow dashed line).

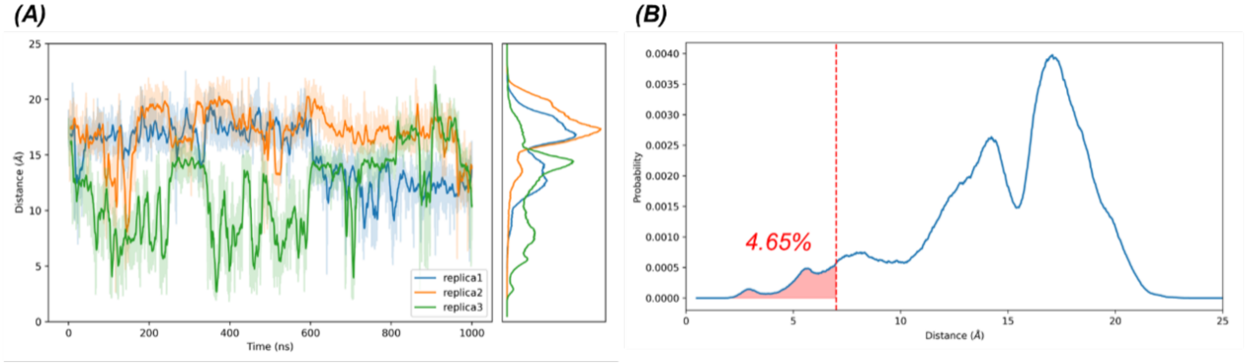


**Figure S27.** (A) Minimum distance between active site (E11, D20, and T26) and PEG polymer along the simulations of **6**. Distribution of each is shown in the right panel. (B) Averaged distance distribution of three replicas, where 4.65% of the frames are considered having interaction between active site and PEG polymer (Minimum distance < 7.0 Å).

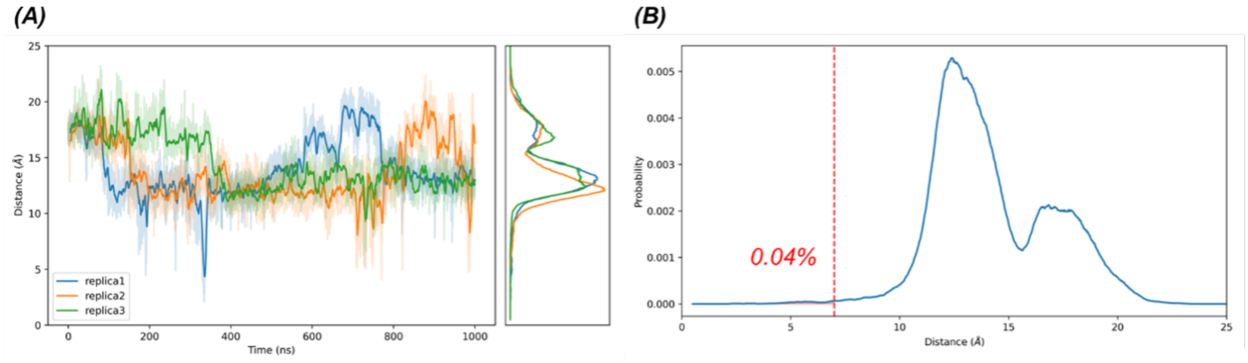


**Figure S28.** (A) Minimum distance between active site (E11, D20, and T26) and PEG polymer along the simulations of **5**. Distribution of each replica is shown in the right panel. (B) Averaged distance distribution of three replicas, where 0.04% of the frames are considered having interaction between active site and PEG polymer (Minimum distance < 7.0 Å).

# References

(1) Kunkel, G. E.; Treacy, J. W.; Montgomery, H. R.; Puente, E. G.; Doud, E. A.; Spokoyny, A. M.; Maynard, H. D. Efficient End-Group Functionalization and Diblock Copolymer Synthesis *via* Au(III) Reagents *Chem. Commun.* **2024**, 10.1039.D3CC05350D. https://doi.org/10.1039/D3CC05350D.

(2) Doud, E. A.; Tilden, J. A. R.; Treacy, J. W.; Chao, E. Y.; Montgomery, H. R.; Kunkel, G. E.; Olivares, E. J.; Adhami, N.; Kerr, T. A.; Chen, Y.; Rheingold, A. L.; Loo, J. A.; Frost, C. G.; Houk, K. N.; Maynard, H. D.; Spokoyny, A. M. Ultrafast Au(III)-Mediated Arylation of Cysteine. *J. Am. Chem. Soc.* **2024**, *146* (18), 12365–12374. https://doi.org/10.1021/jacs.3c12170.

(3) Lundgren, R. J.; Sappong-Kumankumah, A.; Stradiotto, M. A General Catalyst System for Amination of Aryl Chlorides. *Synfacts* **2010**, *2010* (05), 0580–0580. https://doi.org/10.1055/s-0029-1219677.

(4) Sharma, S.; Ntetsikas, K.; Ladelta, V.; Bhaumik, S.; Hadjichristidis, N. Well-Defined Cyclic Polymer Synthesis via an Efficient Etherification-Based Bimolecular Ring-Closure Strategy. *Polym. Chem.* **2021**, *12* (45), 6616–6625. https://doi.org/10.1039/D1PY01337H.

(5) Lorenzo, M. M.; Decker, C. G.; Kahveci, M. U.; Paluck, S. J.; Maynard, H. D. Homodimeric Protein–Polymer Conjugates via the Tetrazine–Trans-Cyclooctene Ligation. *Macromolecules* **2016**, *49* (1), 30–37. https://doi.org/10.1021/acs.macromol.5b02323.

(6) Amber 2022.Bib.D.A. Case, H. M. A., K. Belfon, I.Y. Ben-Shalom, J.T. Berryman, S.R. Brozell, D.S. Cerutti, T.E. Cheatham, III, G.A. Cisneros, V.W.D. Cruzeiro, T.A. Darden, N. Forouzesh, G. Giambaşu, T. Giese, M.K. Gilson, H. Gohlke, A.W. Goetz, J. Harris, S. Izadi, S.A. Izmailov, K. Kasavajhala, M.C. Kaymak, E. King, A. Kovalenko, T. Kurtzman, T.S. Lee, P. Li, C. Lin, J. Liu, T. Luchko, R. Luo, M. Machado, V. Man, M. Manathunga, K.M. Merz, Y. Miao, O. Mikhailovskii, G. Monard, H. Nguyen, K.A. O'Hearn, A. Onufriev, F. Pan, S. Pantano, R. Qi, A. Rahnamoun, D.R. Roe, A. Roitberg, C. Sagui, S. Schott-Verdugo, A. Shajan, J. Shen, C.L. Simmerling, N.R. Skrynnikov, J. Smith, J. Swails, R.C. Walker, J. Wang, J. Wang, H. Wei, X. Wu, Y. Wu, Y. Xiong, Y. Xue, D.M. York, S. Zhao, Q. Zhu, and P.A. Kollman, Amber 2023, University of California, San Francisco. **2023**.

(7) Case, D. A.; Aktulga, H. M.; Belfon, K.; Cerutti, D. S.; Cisneros, G. A.; Cruzeiro, V. W.

D.; Forouzesh, N.; Giese, T. J.; Götz, A. W.; Gohlke, H.; Izadi, S.; Kasavajhala, K.; Kaymak, M. C.; King, E.; Kurtzman, T.; Lee, T.-S.; Li, P.; Liu, J.; Luchko, T.; Luo, R.; Manathunga, M.; Machado, M. R.; Nguyen, H. M.; O'Hearn, K. A.; Onufriev, A. V.; Pan, F.; Pantano, S.; Qi, R.; Rahnamoun, A.; Risheh, A.; Schott-Verdugo, S.; Shajan, A.; Swails, J.; Wang, J.; Wei, H.; Wu, X.; Wu, Y.; Zhang, S.; Zhao, S.; Zhu, Q.; Cheatham, T. E. I.; Roe, D. R.; Roitberg, A.; Simmerling, C.; York, D. M.; Nagan, M. C.; Merz, K. M. Jr. AmberTools. *J. Chem. Inf. Model.* **2023**, *63* (20), 6183–6191. https://doi.org/10.1021/acs.jcim.3c01153.

(8) Banatao, D. R.; Cascio, D.; Crowley, C. S.; Fleissner, M. R.; Tienson, H. L.; Yeates, T. O. An Approach to Crystallizing Proteins by Synthetic Symmetrization. *Proc. Natl. Acad. Sci.* **2006**, *103* (44), 16230–16235. https://doi.org/10.1073/pnas.0607674103.

(9) Fährrolfes, R.; Bietz, S.; Flachsenberg, F.; Meyder, A.; Nittinger, E.; Otto, T.; Volkamer, A.; Rarey, M. ProteinsPlus: A Web Portal for Structure Analysis of Macromolecules. *Nucleic Acids Res.* **2017**, *45* (Web Server issue), W337–W343. https://doi.org/10.1093/nar/gkx333.

(10) Bietz, S.; Urbaczek, S.; Schulz, B.; Rarey, M. Protoss: A Holistic Approach to Predict Tautomers and Protonation States in Protein-Ligand Complexes. *J. Cheminformatics* **2014**, *6* (1), 12. https://doi.org/10.1186/1758-2946-6-12.

(11) Götz, A. W.; Williamson, M. J.; Xu, D.; Poole, D.; Le Grand, S.; Walker, R. C. Routine Microsecond Molecular Dynamics Simulations with AMBER on GPUs. 1. Generalized Born. *J. Chem. Theory Comput.* **2012**, *8* (5), 1542–1555. https://doi.org/10.1021/ct200909j.

(12) Salomon-Ferrer, R.; Götz, A. W.; Poole, D.; Le Grand, S.; Walker, R. C. Routine Microsecond Molecular Dynamics Simulations with AMBER on GPUs. 2. Explicit Solvent Particle Mesh Ewald. *J. Chem. Theory Comput.* **2013**, *9* (9), 3878–3888. https://doi.org/10.1021/ct400314y.

(13) Tian, C.; Kasavajhala, K.; Belfon, K. A. A.; Raguette, L.; Huang, H.; Migues, A. N.; Bickel, J.; Wang, Y.; Pincay, J.; Wu, Q.; Simmerling, C. ff19SB: Amino-Acid-Specific Protein Backbone Parameters Trained against Quantum Mechanics Energy Surfaces in Solution. *J. Chem. Theory Comput.* **2020**, *16* (1), 528–552. https://doi.org/10.1021/acs.jctc.9b00591.

(14) Izadi, S.; Anandakrishnan, R.; Onufriev, A. V. Building Water Models: A Different Approach. *J. Phys. Chem. Lett.* **2014**, *5* (21), 3863–3871. https://doi.org/10.1021/jz501780a.

(15) Ditchfield, R.; Hehre, W. J.; Pople, J. A. Self-Consistent Molecular-Orbital Methods. IX. An Extended Gaussian-Type Basis for Molecular-Orbital Studies of Organic Molecules. *J. Chem. Phys.* **1971**, *54* (2), 724–728. https://doi.org/10.1063/1.1674902.

(16) Hehre, W. J.; Ditchfield, R.; Pople, J. A. Self—Consistent Molecular Orbital Methods. XII. Further Extensions of Gaussian—Type Basis Sets for Use in Molecular Orbital Studies of Organic Molecules. *J. Chem. Phys.* **1972**, *56* (5), 2257–2261. https://doi.org/10.1063/1.1677527.

(17) Bayly, C. I.; Cieplak, P.; Cornell, W.; Kollman, P. A. A Well-Behaved Electrostatic Potential Based Method Using Charge Restraints for Deriving Atomic Charges: The RESP Model. *J. Phys. Chem.* **1993**, *97* (40), 10269–10280. https://doi.org/10.1021/j100142a004.

(18) Besler, B. H.; Merz Jr., K. M.; Kollman, P. A. Atomic Charges Derived from Semiempirical Methods. *J. Comput. Chem.* **1990**, *11* (4), 431–439. https://doi.org/10.1002/jcc.540110404.

(19) Singh, U. C.; Kollman, P. A. An Approach to Computing Electrostatic Charges for Molecules. *J. Comput. Chem.* **1984**, *5* (2), 129–145. https://doi.org/10.1002/jcc.540050204.

(20) Frisch, M. J.; Trucks, G. W.; Schlegel, H. B.; Scuseria, G. E.; Robb, M. A.; Cheeseman, J. R.; Scalmani, G.; Barone, V.; Petersson, G. A.; Nakatsuji, H.; Li, X.; Caricato, M.; Marenich, A. V.; Bloino, J.; Janesko, B. G.; Gomperts, R.; Mennucci, B.; Hratchian, H. P.; Ortiz, J. V.;

Izmaylov, A. F.; Sonnenberg, J. L.; Williams; Ding, F.; Lipparini, F.; Egidi, F.; Goings, J.; Peng, B.; Petrone, A.; Henderson, T.; Ranasinghe, D.; Zakrzewski, V. G.; Gao, J.; Rega, N.; Zheng, G.; Liang, W.; Hada, M.; Ehara, M.; Toyota, K.; Fukuda, R.; Hasegawa, J.; Ishida, M.; Nakajima, T.; Honda, Y.; Kitao, O.; Nakai, H.; Vreven, T.; Throssell, K.; Montgomery Jr., J. A.; Peralta, J. E.; Ogliaro, F.; Bearpark, M. J.; Heyd, J. J.; Brothers, E. N.; Kudin, K. N.; Staroverov, V. N.; Keith, T. A.; Kobayashi, R.; Normand, J.; Raghavachari, K.; Rendell, A. P.; Burant, J. C.; Iyengar, S. S.; Tomasi, J.; Cossi, M.; Millam, J. M.; Klene, M.; Adamo, C.; Cammi, R.; Ochterski, J. W.; Martin, R. L.; Morokuma, K.; Farkas, O.; Foresman, J. B.; Fox, D. J. Gaussian 16 Rev. C.01, 2016.

(21) Darden, T.; York, D.; Pedersen, L. Particle Mesh Ewald: An N·log(N) Method for Ewald Sums in Large Systems. *J. Chem. Phys.* **1993**, *98* (12), 10089–10092. https://doi.org/10.1063/1.464397.

(22) Onufriev, A.; Bashford, D.; Case, D. A. Exploring Protein Native States and Large-Scale Conformational Changes with a Modified Generalized Born Model. *Proteins* **2004**, *55* (2), 383–394. https://doi.org/10.1002/prot.20033.

(23) Pettersen, E. F.; Goddard, T. D.; Huang, C. C.; Meng, E. C.; Couch, G. S.; Croll, T. I.; Morris, J. H.; Ferrin, T. E. UCSF ChimeraX: Structure Visualization for Researchers, Educators, and Developers. *Protein Sci. Publ. Protein Soc.* **2021**, *30* (1), 70–82. https://doi.org/10.1002/pro.3943.

(24) Humphrey, W.; Dalke, A.; Schulten, K. VMD: Visual Molecular Dynamics. *J. Mol. Graph.* **1996**, *14* (1), 33–38, 27–28. https://doi.org/10.1016/0263-7855(96)00018-5.
